# Coupled air–sea interactions drove and sustained the 2013–2016 North Pacific marine heatwave

**DOI:** 10.1038/s41467-026-76096-0

**Published:** 2026-08-04

**Authors:** Wenrui Jiang, Gaël Forget, Yuanyuan Song, Thomas W. N. Haine

**Affiliations:** 1 Department of Earth and Planetary Sciences, Johns Hopkins University, Baltimore, MD USA; 2 Department of Earth, Atmospheric and Planetary Sciences, Massachusetts Institute of Technology, Cambridge, MA USA

**Keywords:** Atmospheric dynamics, Physical oceanography, Physical oceanography

## Abstract

The 2013–2016 marine heatwave (MHW) in the Northeastern Pacific ranks among the most intense extratropical ocean warming events on record. Its intricate evolution has complicated efforts to understand the underlying dynamics crucial for predicting future MHWs. Here, we track the MHW using a closed, three-dimensional Lagrangian heat budget that quantifies the processes driving its evolution. Three-dimensional particle trajectories separate the MHW into northern and southern components and reveal that they are of distinct kinematic origin. We quantitatively demonstrate through a closed budget that advection, rather than surface forcing, plays the dominant role in sustaining the MHW’s heat content. The northern component was maintained by a weakening of Ekman heat transport across the North Pacific Current, combined with reduced wintertime surface heat loss. The southern component was driven by weakened Ekman upwelling caused by reduced alongshore winds over the California Current. We further identify a persistent low sea-level pressure anomaly over the central Pacific that altered wind patterns, redistributing heat and moisture poleward to ultimately force both parts of the MHW. Elevated surface temperatures, in turn, amplified this pressure anomaly, completing a positive feedback loop that sustained the event across multiple years.

## Abstract (web summary)

Using 3D particle tracking, this study shows that weakened ocean currents—not surface heating—mainly sustained the 2013–2016 Pacific marine heatwave, as part of a positive air-sea feedback loop.

## Introduction

A significant increase in sea surface temperature (SST) emerged during the winter of 2013–2014^1^, evolving into a record-breaking marine heatwave (MHW) in the Northeast Pacific (NEP) Ocean that persisted until early 2016^2^. Commonly nicknamed the blob, this event extended from the Gulf of Alaska (GOA) to the California Current region (CC), with peak SST anomalies exceeding 3 °C^2,3^. The intensity and persistence of the MHW led to widespread ecological disruption^4^, including extensive kelp forest loss along the California coast^5,6^, a large-scale toxic algal bloom^7^, and elevated whale entanglement rates^8^, resulting in economic losses of hundreds of millions of dollars^9^.

Although MHWs are typically defined by SST anomalies, recent studies have highlighted the importance of tracking heat anomalies in three spatial dimensions (3D) over time^10–12^. Subsurface temperature extremes often persist longer than their surface counterparts and contribute to the overall longevity of a MHW^13–16^. For example, the 2019-2020 NEP MHW was largely confined to the top 50 m and lasted less than a year^17^, whereas the 2013-2016 warm anomaly extended beyond 150 m depth and persisted for more than two years^16^. Such deep penetration also emphasizes the role of ocean heat transport relative to surface heat flux^18,19^, underscoring the need for a 3D Lagrangian view of the MHW life cycle^10,11,20^.

Under anthropogenic climate change, MHWs have become more frequent and intense in recent decades^21–24^. Each region and event is driven by a distinct combination of changes in surface heat fluxes, ocean mixing, and transport processes^12,24,25^. To better understand the evolution of an MHW, it is crucial to diagnose the timing, location, and relative importance of these drivers. However, such diagnostic analyses remain challenging with existing approaches.

Remote atmospheric teleconnections originating from the tropics^3^, mid-latitudes^26^, and the Arctic^23^ have been shown to create favorable atmospheric conditions for ocean warming in the NEP. Previous studies have also shown that SST anomalies in the NEP can trigger remote atmospheric responses, including droughts^27,28^ and cold winters^29–32^. Yet, the role of local atmospheric feedbacks—through which SST anomalies modulate their own drivers—remains largely unexamined.

In this work, we track the 3D kinematics of MHW watermasses rather than just SST patterns. Existing analyses of the Blob have largely focused on the onset of the event in late 2013^1,23,26,33^, examined only its surface signature^25,34^, or primarily investigated either atmospheric^35^ or oceanic^12^ drivers in isolation—providing only a partial account of how the MHW evolved throughout its full life cycle. By tracking the evolving 3D footprint of the warm anomaly rather than its surface expression alone, our framework captures the fundamentally advective nature of this large-scale propagating event and quantifies where, when, and by how much each process—including surface fluxes, advection, and mixing—modifies the heat content of the MHW at every stage of its life cycle. Our analyses are based on the Estimating the Circulation and Climate of the Ocean, Version 4 Release 4 (ECCO4) global ocean state estimate^36,37^, whose closed heat budgets and data-constrained flow fields make such a Lagrangian accounting of observed MHWs possible. Crucially, ECCO4 also allows us to examine both atmospheric and oceanic dynamics within a single, self-consistent framework and resolves the coupled air-sea interactions that sustained the event.

## Results

**Fig. 1:**
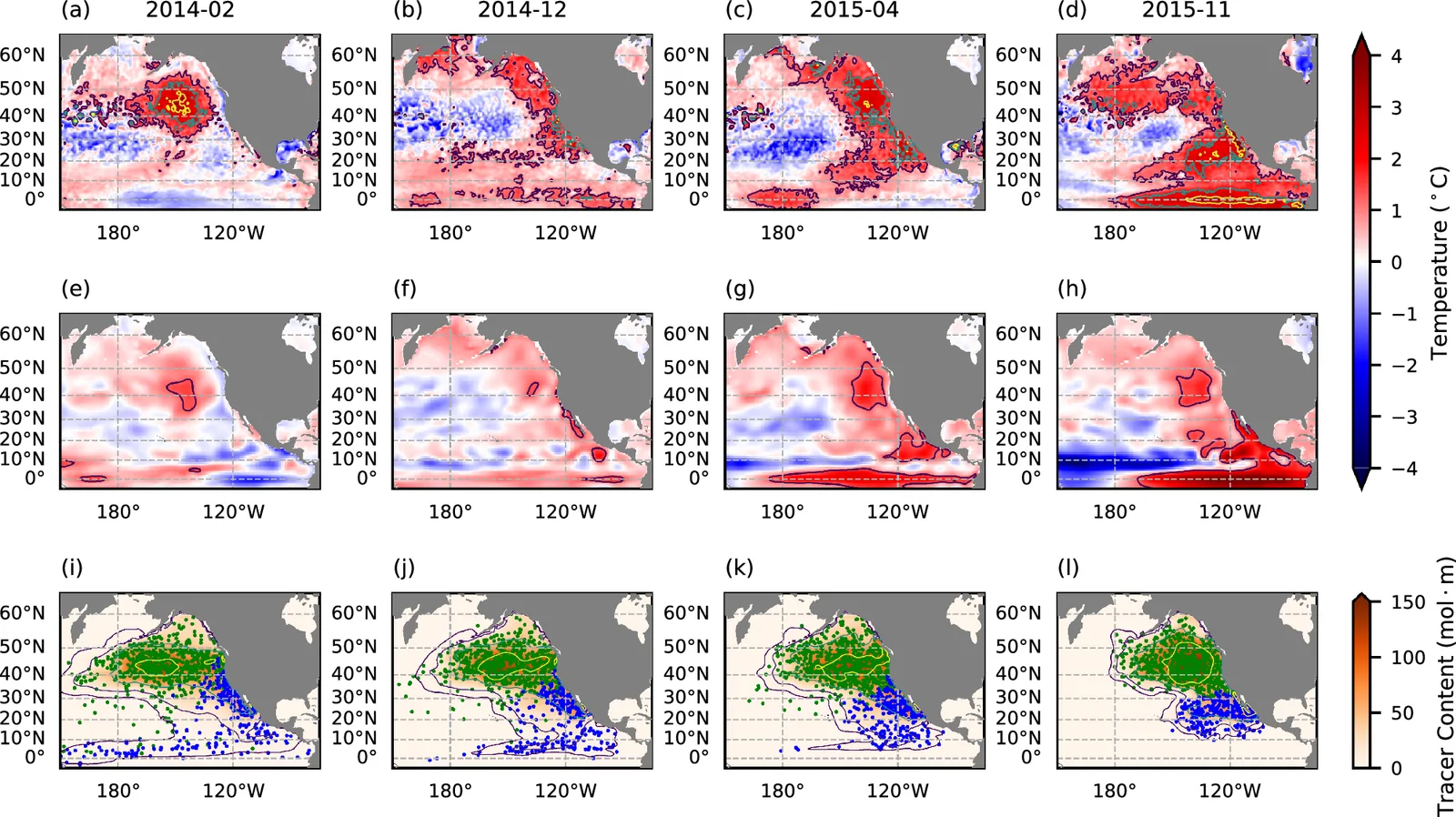
Temporal evolution of the Northeast Pacific (NEP) marine heat wave (MHW). **a–d** Monthly-averaged sea surface temperature (SST) anomaly from the Optimum Interpolation Sea Surface Temperature product (OISST), **e–h** monthly-averaged upper 150 m temperature anomaly from the Estimating the Circulation and Climate of the Ocean, Version 4 Release 4 product (ECCO4), and **i–l** snapshots of vertically-integrated passive tracer concentration being tracked backwards in time from release on March 1, 2016. Contour levels for (**a–d**) are 1 ° C (purple), 2 ° C (green), and 3 ° C (yellow); for (**e–h**), the contour level is 1.25 ° C. Contours in (**i–l**) denote integrated tracer concentrations of 1 mol ⋅ m (dark purple), 10 mol ⋅ m (purple), 50 mol ⋅ m (green), and 100 mol ⋅ m (yellow). **i–l** also show a (randomly-selected) subset of Lagrangian particle locations being tracked backwards in time; northern part of the MHW (N-MHW) particles are green, and southern part of the MHW (S-MHW) particles are blue.

The evolution of the MHW SST anomaly is shown in Fig. 1a–d, based on the Optimum Interpolation Sea Surface Temperature (OISST) dataset (see also Figs. S.1, 2). A warm SST anomaly first emerged in the winter of 2013 (Figs. S.1a–c, 2a–c). In early 2014, an SST anomaly north of 30° N reached its peak, and another warm anomaly gradually developed along the California Current (Fig. 1a, b). The northern anomaly persisted until a relatively cool phase from autumn 2014 through spring 2015, followed by a period of rapid warming. Meanwhile, the southern anomaly continued to intensify and expand offshore (Fig. 1c, d). Both anomalies eventually weakened in early 2016 (Figs. S.1o, p, 2o, p). The differences in timing and spatial separation between the northern and southern anomalies suggest they are likely influenced by distinct mechanisms.

**Fig. 2:**
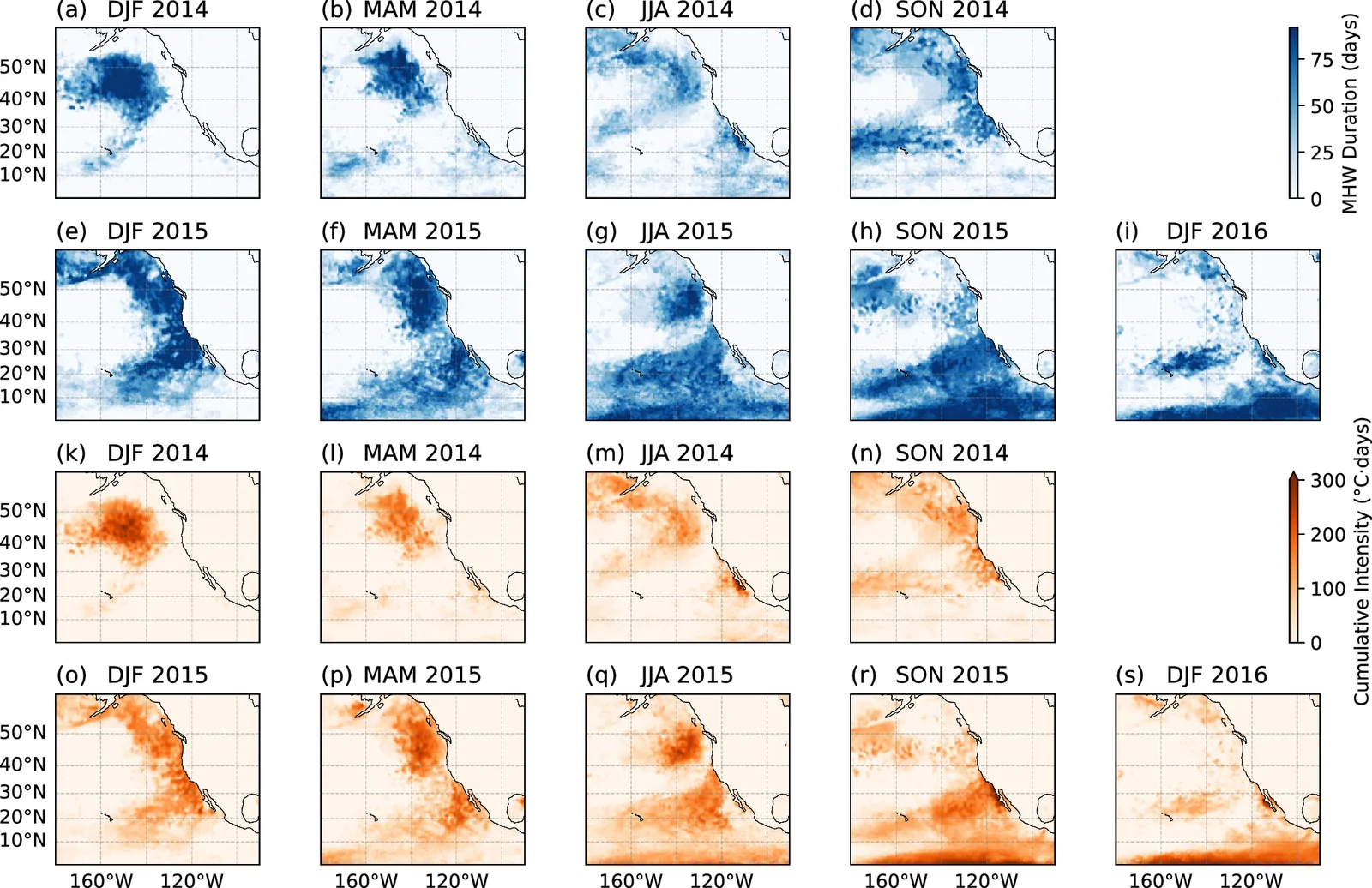
Seasonal marine heat wave (MHW) duration and cumulative intensity from the Optimum Interpolation Sea Surface Temperature product (OISST). **a–i** The MHW duration in each season between December 1st, 2013 and March 1st, 2016. **k–s** The MHW cumulative intensity in the same periods of time.

To quantify the MHW’s statistics, we use daily OISST data to calculate two complementary metrics: duration and cumulative intensity (Fig. 2, see Methods). In every season from December 2013 to February 2016, large parts of the NEP exhibit MHW conditions more than half the time (~45 days) and the cumulative intensity routinely exceeds 100 ° C days. Some areas even show MHW conditions for almost the entire season (~90 days) with cumulative intensities exceeding 300 ° C days. For comparison, durations of 16 days and cumulative intensities of 50 ° C days have been recently identified as critical MHW thresholds to trigger fishing ground contraction^38^.

Given that the MHW penetrated deep into the ocean and carried substantial thermal inertia^14,16^, an analysis of upper ocean heat content (OHC) provides a more complete characterization than SST alone^15,39,40^. The surface temperature in ECCO agrees well with OISST (Figs. S.1, 2). The interior upper-ocean temperature also agrees well with in situ measurements from Argo^41^ (Figs. S.3, S.4, see also refs. ^12,36,37,42^), which justifies the assessment of the subsurface temperature anomaly averaged over the top 150 m (Fig. 1E–H). Although it closely correlates with SST, the averaged OHC anomaly more clearly reveals eastward propagation with the general circulation (Fig. S.5). To investigate the nature of this propagation, Fig. 1i–l (see also Figs. S.6–8) displays the concentration of a passive tracer and the locations of Lagrangian particles that were injected into the MHW (see Methods) and advected eastward along the North Pacific Current (NPC). The center of the tracer distribution (yellow contours) moves eastward faster than the center of the depth-integrated temperature anomaly (black contours in Fig. 1e–h, see also Supplementary Movie 1). This shows that the MHW is neither stationary nor purely Lagrangian.

The water masses represented by the particles injected north (south) of 30° N are referred to as N-MHW (S-MHW). Minimal mixing between N-MHW (green particles) and S-MHW (blue particles) demonstrates the lack of a shared origin (Fig. 1i–l), and again, suggests distinct life cycles that further motivate separate Lagrangian budget analyses. Notably, N-MHW and S-MHW represent time-evolving Lagrangian volumes that record the history of water parcels, which differ conceptually from common definitions of MHWs based on an instantaneous SST threshold^40^. Heat budget results calculated within N-MHW and S-MHW are also conceptually different from those defined on a surface mixed-layer or a fixed Eulerian volume.

### Lagrangian budget attribution

**Fig. 3:**
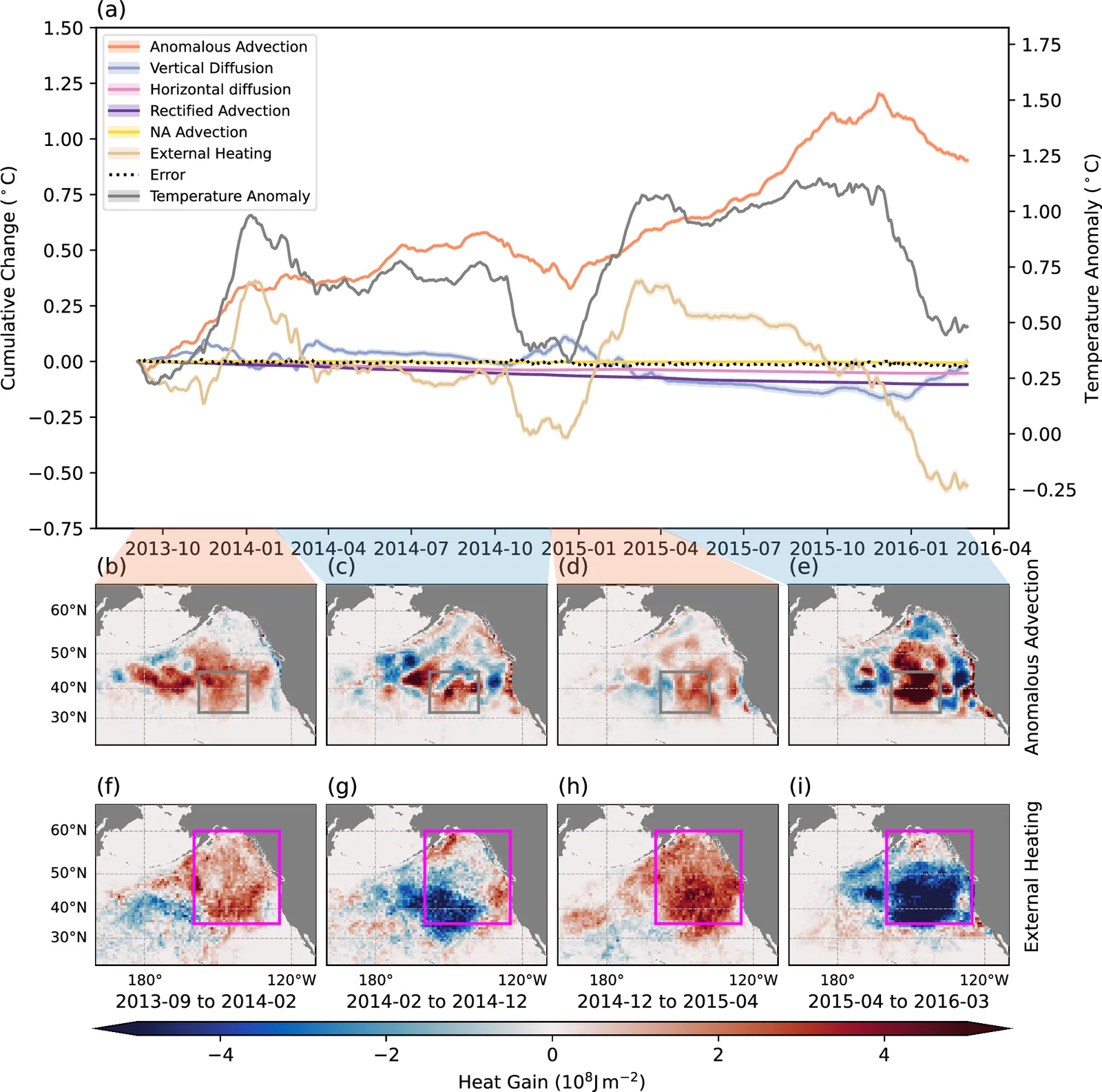
Lagrangian heat budget of the northern part of the marine heat wave (N-MHW). **a** Time series of particle-averaged temperature anomaly (gray line) and cumulative contribution from each right-hand-side term of Equation (3). The spread area around each line shows the range of plus or minus one standard error for the sample mean. The dotted line shows the residual incurred during budget analysis. **b–i** Map of contribution from anomalous advection (**b–e**) and surface heating (**f–i**) in the four periods, which are highlighted by red (growth) and blue (maturity/decay) polygons. The gray box in (**b–e**) denotes the longitude-latitude region spanning 158° W to 138° W and 32° N to 45° N. The magenta box in (**f–i**) covers the area from 125° W to 160° W and 35° N to 60° N.

**Fig. 4:**
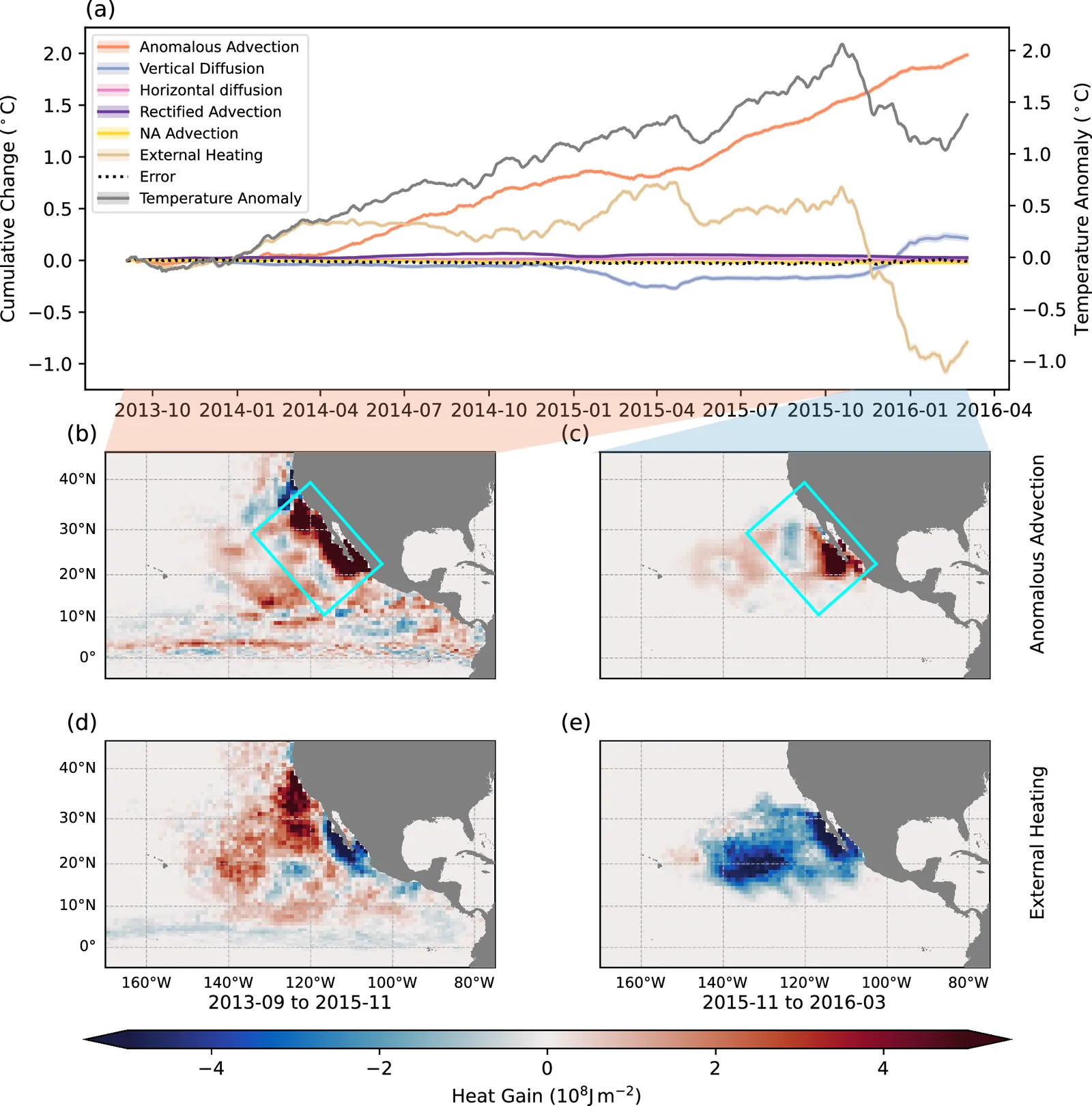
Lagrangian heat budget of the southern part of the marine heat wave (S-MHW). **a** Time series of particle averaged temperature anomaly and cumulative contribution from each right-hand-side term of Equation (3) for S-MHW, similar to Fig. 3a. **b**, **c** Spatial map of contribution from anomalous advection and (**d**, **e**) surface heating similar to Fig. 3b, i. The cyan box in (**b**, **c**) denotes the California Current (CC) upwelling zone, with corners at (120° W, 39. 5° N), (102. 5° W, 22. 5° N), (116. 6° W, 10.55° N), and (134. 1° W, 29. 2° N).

The Lagrangian heat budget (see Methods Section 4.5) quantifies relative contributions of surface heating, advection, and diffusion (Figs. 3–4). Similar analyses from an Eulerian perspective can be found in Fig. S.9. The N-MHW experienced persistently high temperature during two periods: December 2013 to October 2014, and March 2015 to November 2015 (Fig. 3a). Temperature variability operates on different timescales. On shorter timescales (e.g., a few months, as seen between December 2014 and April 2015), temperature variability is strongly influenced by anomalous surface heating (${{{\mathcal{Q}}}}^{\prime}$, due to surface turbulent and radiative fluxes). ${{{\mathcal{Q}}}}^{\prime}$ is responsible for both rapid warming and rapid cooling events (late 2014 and early 2016) that strongly damp the temperature anomaly. On longer timescales, however, anomalous advection ($-{\mathbb{A}}(U^{\prime},\overline{\Theta })$, advection by anomalous velocity across mean temperature gradients) acts to sustain the accumulated heat, while temporal fluctuations in surface heating tend to average out. Until early 2016, anomalous advection remained consistently positive, sustaining the heat content anomaly and offsetting the cooling effect of surface fluxes. This pattern persisted through the maturity/decay periods (shown as blue-shaded periods in Fig. 3a), when temperature anomalies were steady or decreasing. Vertical diffusion is strongly anticorrelated with surface heating, redistributing heat from the surface to the ocean interior. Overall, vertical diffusion and other processes play minor roles compared to anomalous advection and surface heating.

Figure 3b–i show how anomalous advection and surface heating affect the N-MHW spatially during each growth (predominantly warming) or maturity/decay (steady or cooling) periods. Within the GOA (highlighted by the gray box), anomalous advection provides constant localized heating over both growth (red-shaded) and maturity/decay (blue-shaded) periods (Fig. 3b–e). In contrast, surface heating exhibits greater spatial uniformity but stronger temporal variability, with widespread heating during growth periods (Fig. 3f, h) and cooling over maturity/decay periods (Fig. 3g, i). Noticeably, the widespread decrease in seasonal heat loss drove the sharp anomalous warming during the winter of 2014–2015, as shown in Fig. 3a, h.

Similar to the N-MHW, anomalous advection and surface heating contribute most to the evolution of the warm anomaly in S-MHW (Fig. 4a). Damping by ${{{\mathcal{Q}}}}^{\prime}$ again dominated the decay phase after November 2015 (blue-shaded). However, during the growth phase before November 2015 (red-shaded), ${{{\mathcal{Q}}}}^{\prime}$ played a much smaller role in S-MHW than in N-MHW, while anomalous advection accounted for most of the heat content increase. This warming was strongly concentrated in the CC upwelling zone (oblique cyan boxes in Fig. 4b, c). As shown in Fig. 4d, surface heating warmed the ocean north of 30° N while cooling occurred south of it. This spatial cancellation of surface heating resulted in a relatively weak net contribution to the S-MHW.

### Prolonged heat accumulation by anomalous Ekman transport

**Fig. 5:**
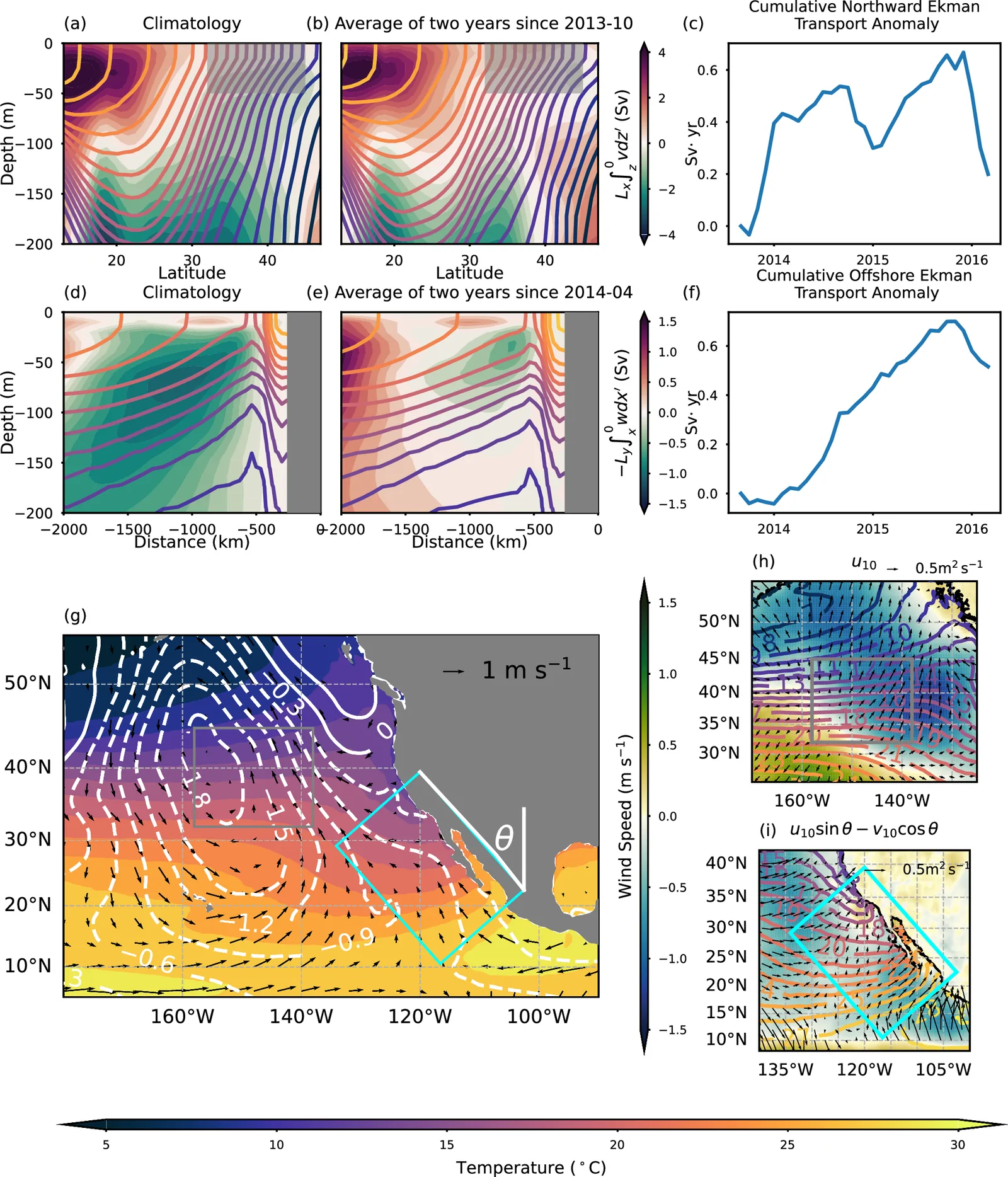
Diagnostics of warming due to anomalous advection. **a**, **b** Time-mean proxy stream function (shading; see Methods) and temperature contours (with 1 ° C spacing) over (**a**) the whole Estimating the Circulation and Climate of the Ocean, Version 4 Release 4 (ECCO4) period (climatology) and (**b**) October 2013 to September 2015. The gray box indicates the same region defined in Fig. 3b–e in the upper 50 m. **c** Cumulative northward Ekman transport anomaly across 40° N, between 138° W and 158° W. **d**, **e** Time-mean proxy stream function (shading; see Methods) and temperature contours over (**d**) the whole ECCO4 period and (**e**) April 2014 to March 2016. **f** Cumulative offshore Ekman transport anomaly in the California Current (CC) across the midline. **g** Anomalous 10 m wind (arrows) and sea level pressure (SLP; contours, in hPa) averaged over 2014-2015, superimposed on climatological mean sea surface temperature (SST; shading). **h**, **i** Anomalous 10 m zonal (**h**) and alongshore (**i**) wind (shadings), the vertically integ rated Ekman velocity vectors (arrows), and the climatological mean SST over the ECCO4 period (contours, with 1 ° C spacing). Quantities plotted in (**h**) and (**i**) are averaged over two-year periods since October 2013 and April 2014, respectively. **a–f** are based on ECCO4, while (**g–i**) are derived from ECMWF Reanalysis v5 (ERA5).

Persistent accumulation of heat from anomalous advection in the GOA (gray box in Fig. 3b–e) contributes to the growth of N-MHW and offsets surface cooling (e.g., Fig. 3g, i). The definition of the GOA region closely aligns with the SST front along the NPC, as shown in the gray box in Fig. 5a, b, where a steep meridional temperature gradient is evident from tightly packed isotherms.

The shading in Fig. 5a, b represents a proxy overturning stream function (see Methods section 4.6 and Fig. S.10–S.11), whose vertical derivative reflects the meridional velocity. The persistent heating from anomalous advection occurred mainly during the two-year period starting October 2013. Over this period, the thermal structure remained qualitatively similar to the climatology (Fig. 5a, b). However, the proxy overturning stream function indicates a pronounced contraction of the Ekman-driven overturning cell^43^ beneath the gray-shaded region. Under normal conditions, eastward winds induce equatorward Ekman transport, which cools the GOA surface^43^. This transport was substantially reduced (less negative) during the study period, leading to anomalous warming through advection. The reduction in equatorward Ekman transport—approximately 0.3 Sv (Fig. 5c)—is comparable in magnitude to the observed decline in the proxy stream function in the upper 50 m (Fig. 5b). Weakened surface zonal wind velocity (and associated wind stress) drives this reduced equatorward transport (Fig. 5h).

Pronounced warming of S-MHW was confined to the coastline, beginning in April 2014 and continuing through mid-2016. As shown in Fig. 5d, under normal conditions, southward alongshore winds generate offshore Ekman transport, inducing strong upwelling that brings cold subsurface water to the surface, thereby cooling the upper ocean^44^. During the two years since April 2014, this overturning cell in the CC region was substantially weakened (Fig. 5e). The cell carried about 1 Sv less transport and became vertically confined to the upper 100 m. Anomalous onshore transport (i.e., a reduction in offshore flow) depicted in Fig. 5f was attributed to a decrease in southward alongshore wind stress (Fig. 5i). The weakened offshore transport led to diminished upwelling and, consequently, a reduction in cooling. As surface temperatures rose due to reduced upwelling, vertical stratification increased (Fig. 5d, e), further inhibiting upwelling and compounding the warming effect^44^.

Figure 5g connects the averaged sea level pressure (SLP) anomaly with the anomalous surface wind patterns responsible for the Ekman-driven warming (i.e., reduced cooling) in both the GOA and CC regions. This large-scale SLP anomaly ( ~ 5000 km in diameter, exceeding the typical Rossby radius^45^) generated the anomalous wind in both GOA and CC via geostrophic balance. Thus, this SLP anomaly ultimately drove the warming observed in both the N-MHW and S-MHW regions.

### Wintertime heating of anomalous surface heat flux

**Fig. 6:**
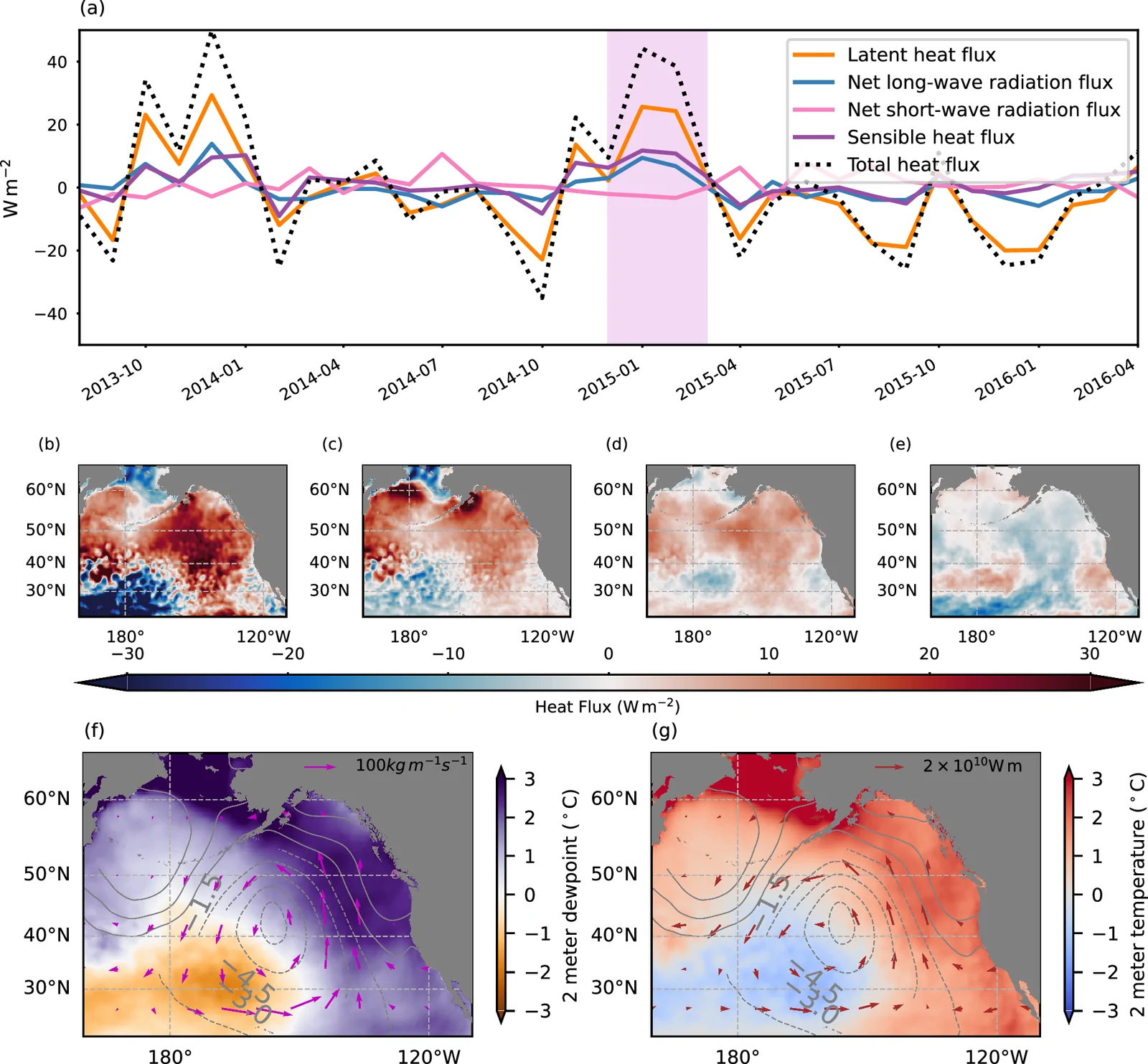
Diagnostics of warming due to surface heat fluxes. **a** Time series of anomalous surface heat fluxes, averaged in the northern marine heat wave (N-MHW) magenta box defined in Fig. 3f–i, including longwave radiation, shortwave radiation, sensible, latent heat, and their total. **b–e** Anomalous surface heat fluxes averaged from December 2014 to January 2015 (highlighted period in (**a**)). **f**, **g** Anomalous (**f**) 2-meter dewpoint temperature and (**g**) 2-meter temperature averaged over the same period (shadings). The vertically integrated water vapor flux and heat flux anomalies are shown as arrows in (**f**) and (**g**), overlaid on sea level pressure (SLP) anomaly (contours with 0.3 hPa spacing).

While anomalous advection contributes to prolonged accumulation of heat, surface heating plays an important role in wintertime strengthening of N-MHW (Fig. 3a). The net surface heat flux in ECMWF Reanalysis v5 (ERA5) clearly highlights the two warming episodes during the winters of 2013–2014 (Fig. S.12) and 2014–2015 (Fig. 6a). In both events, the anomalous surface warming (i.e., reduced heat loss) was primarily driven by anomalous latent heat flux, sensible heat flux, and longwave radiation, with latent heat emerging as the principal contributor. This subsection focuses on the second N-MHW warming episode in the winter of 2014-2015. Similar analyses for the first warming episode are provided in Figs. S.12 and S.13 (see also ref. ^1^).

In late 2014, SST remained anomalously high, as shown in Fig. 1b. This implies that the reduction in heat loss was likely driven by anomalous atmospheric or radiative conditions. Surface wind speed, air temperature, and humidity (dew point) influence the latent and sensible heat fluxes. Atmospheric cloud cover, temperature, and moisture profiles affect the surface radiative fluxes. Compared with total radiative fluxes, clear-sky conditions exhibit only minor differences, implying that clouds play a limited role in retaining heat (Fig. S.14). The warming region experienced an increase in wind speed (Fig. S.15a), which, in isolation, would enhance surface turbulent heat loss—opposite to the observed decrease—suggesting that the reduction in heat loss is primarily driven by thermodynamic variables.

Figure 6b–g reveals strong spatial correlations among anomalous heat flux, temperature, and humidity patterns. Lateral advection was the primary driver of the anomalous thermodynamic structure over the warming region (Fig. S.15a, c). Strong poleward heat and vapor transport aligns closely with elevated surface air temperature and dew point temperature (Fig. 6f, g), as well as increased column-integrated temperature and vapor content (Fig. S.15b, d). The Aleutian Low was particularly intense and far south during the winter of 2014–2015, as indicated by the negative SLP anomalies (dashed contours) in Fig. 6f, g. Winds associated with this low SLP system transported warm, moist air from lower latitudes into the region of interest, consistent with the lateral flux vectors. These findings suggest that the anomalously strong Aleutian Low was ultimately responsible for the surface warming observed during the winter of 2014–2015.

## Discussion

Using a Lagrangian budget integration method, we demonstrate, both causally and quantitatively, that anomalous oceanic advection across the NPC temperature front and the CC are the primary drivers of the 2013–2016 MHW in the subpolar and subtropical NEP, respectively. While reduced Ekman transport has been qualitatively noted as a contributing factor^1,33^, our Lagrangian budget reveals that the weakening of Ekman heat transport specifically across the NPC temperature front is the single largest contributor to the growth of the N-MHW. Reduction in surface ocean heat loss (i.e., positive anomalous heat fluxes) contributed significantly to the rapid wintertime strengthening of the N-MHW during the winters of 2013–2014 and 2014–2015. By contrast, during the decay phase of the MHW, enhanced surface heat loss acts as the primary mechanism eroding the MHW.

Anomalous heat advection is instrumental to the MHW growth and sustenance both at the GOA temperature front and at the CC upwelling zone (gray and cyan boxes in Figs. 3 and 4, respectively). In both regions, wind stress induces heat transport that leads to heat accumulation (Figs. 3a, 4b, 6c, f). Improved heat transport estimates in these regions are therefore essential for monitoring and predicting future MHWs. In addition, atmospheric conditions in the GOA also strongly influence surface heat flux, through the opening of a corridor for atmospheric heat and moisture flux (Fig. 6). Interestingly, four landfalling atmospheric river events occurred during this period of anomalous warming^46,47^, which are potentially linked to the enhanced heat and moisture flux into the GOA.

A key finding enabled by our Lagrangian framework is that N-MHW and S-MHW do not share a common origin and are kinematically independent. Their concurrence was due to a shared dynamical driver—a low SLP anomaly in the central Pacific. In the GOA, it induced a decrease in zonal wind stress along the mean NPC temperature front that reduced Ekman cooling. In the winter of 2014, this low SLP anomaly (a deep, southward Aleutian Low) further strengthened and moved large amounts of heat and moisture from lower latitudes to the GOA, causing anomalously elevated atmospheric heat and moisture content, which then suppressed surface latent, sensible, and longwave heat loss of the N-MHW. Along the California coast, the SLP low caused a decline in alongshore wind stress, weakening the CC upwelling system, reducing cooling and thereby contributing to the S-MHW. As we argue below, this atmospheric forcing is itself partly sustained by the MHW, suggesting a positive feedback loop.

A warm SST anomaly along the eastern boundary of the Pacific, spanning the latitude range of both the S-MHW and N-MHW, is frequently identified as a signature of the Pacific Decadal Oscillation (PDO)^48,49^, which has also been linked to Aleutian low^50,51^. Despite the similarities, there are important distinctions between the 2013–2016 MHW and typical positive PDO events. In a typical PDO positive phase, a strong and widespread cold anomaly dominates the central Pacific and covers an area larger than the warm anomaly along the eastern boundary^49^. Such a cold anomaly is largely absent in the MHW, only emerging near its end (Fig. S.1), and, in fact, the PDO phase in 2014 is negative^49^. More importantly, the ocean heat content anomaly associated with the MHW greatly exceeds that of typical positive PDO events in both intensity and spatial extent. A typical warm anomaly linked to a positive PDO spans 5–10 degrees of longitude and reaches a peak SST anomaly of  ~ 0. 6 ° C (one standard deviation)^49^, whereas the MHW covers 20–30 degrees of longitude (Fig. 1) and reaches a peak SST anomaly of  ~ 3 ° C. As a result, the MHW exerts a stronger influence on atmospheric conditions.

The large heat inertia of the MHW also means that the transport of the anomaly by the ocean current plays a greater role in the MHW than in a typical PDO event. Dynamically, this introduces the ocean transport time scale and allows non-local feedbacks^18^. For example, the Ekman transport induced heating observed along the NPC occurs upstream of the primary SST anomalies.

**Fig. 7:**
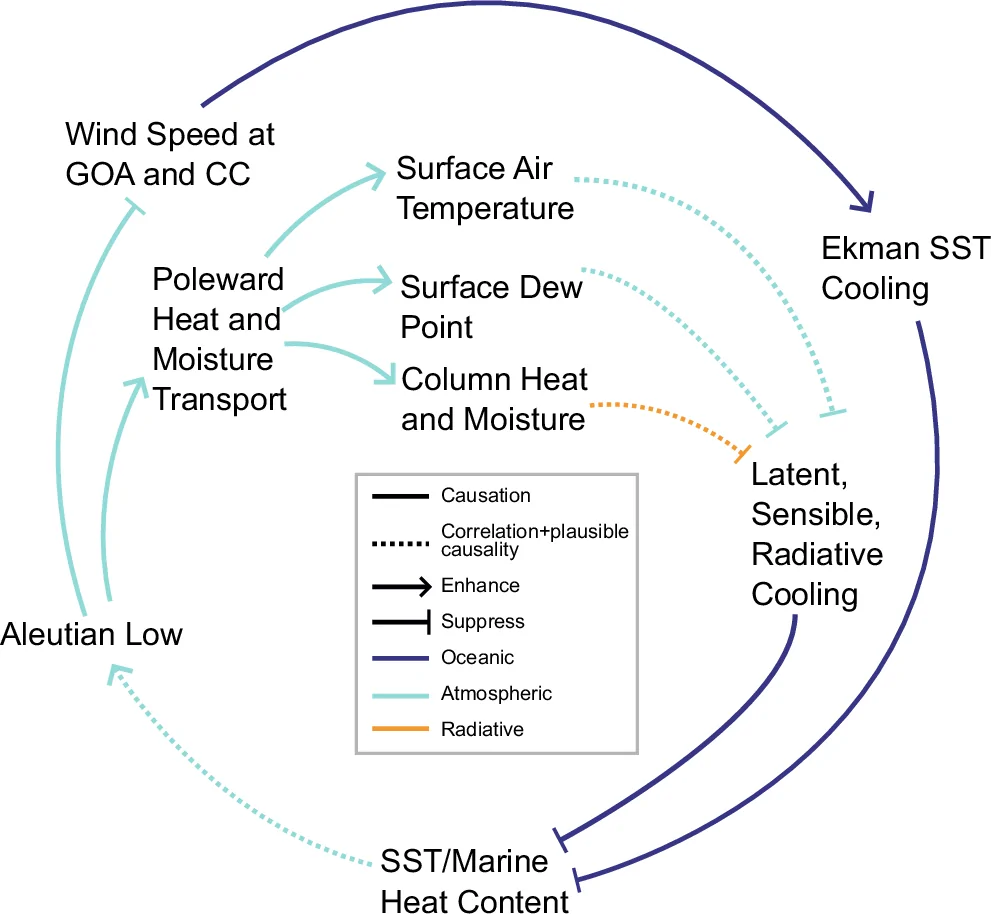
Schematic of the feedback mechanism of marine heat wave (MHW) persistence and enhancement. GOA, CC, and SST stand for Gulf of Alaska, California Current, and sea surface temperature, respectively. The light blue, orange, and dark blue arrows refer to atmospheric, radiative, and oceanic processes, respectively. The dashed arrows denote relations that exhibit strong correlation and plausible mechanism but, to date, remains to be better quantified.

It is plausible that causality between the MHW and the SLP low also operates in the opposite direction: the extensive warm SST anomaly may have contributed to the observed SLP low^52,53^. As shown in Figs. 1 and 6, the peak temperature ( ~ 0. 6 ° C), rate of heat loss ( ~ 20 W/m^2^), and size ( ~ 10^7^ km^2^) of the MHW’s SST anomaly are comparable to El Niño events^54,55^, which are known to induce significant SLP lows above their warm SST anomalies^56^. We hypothesize that the MHW exerts a similar influence on the atmosphere. Indeed, based on reanalysis products, coupled climate models, and atmospheric models forced by prescribed SST, previous works^31,32,57,58^ concluded that the second empirical orthogonal function (EOF) of SST, which has a similar spatial pattern to the MHW, can induce a low pressure in the central Pacific similar to that observed in 2014–2015. While the individual components of this interaction—SST influence on SLP^31,32,57,58^ and atmospheric forcing of oceanic heat content^1,3^—have been studied separately, our Lagrangian framework allows us to close the loop (Fig. 7) quantitatively: at the expense of temporarily losing heat through surface flux (e.g. Fig. 3g), the MHW warms the surface air and reduces SLP; by inducing anomalous Ekman transports and surface fluxes, the low SLP more than compensates for the original heat loss. This completes a positive feedback loop reminiscent of the wind-evaporation-SST feedback^52^, as illustrated in Fig. 7, and offers a mechanistic explanation for the unusual multi-year persistence of the event.

If a positive feedback loop can thus reinforce MHWs in the NEP, one might expect events in 2013–2016 to occur more frequently. The rarity of such events may be explained by two factors. First, while local SST anomalies can significantly influence atmospheric circulation, the strength and position of SLP anomalies are also influenced by other climate modes, including, crucially, the El Nino-Southern Oscillation, remote SST forcing, and the seasonal cycle^3,23,26,57^. Thus, the positive feedback loop efficiency may depend on the timing of the MHW relative to those other climate modes. Second, warm SST anomalies enhance heat loss to the atmosphere via increased evaporation, sensible heat flux, and upward longwave radiation, thus forming a competing negative feedback^52,59^. When the heat anomaly has larger thermal inertia, this damping process operates over a longer timescale^19^. The multi-year persistence of the 2013–2016 MHW likely thus resulted from a combination of favorable timing and an unusually large heat content anomaly.

It is plausible that the negative SST feedback will become less effective in a warmer climate. With increased humidity and greenhouse gas concentrations, longwave radiative damping of warm anomalies becomes less efficient^60^. As a result, the air-sea coupled system may become more effective in amplifying climate fluctuations and driving future extremes.

## Methods

### Data

The main dataset used in this study is the ECCO4 state estimate reanalysis (Estimating the Circulation and Climate of the Ocean, Version 4, release 4), which provides a comprehensive heat budget estimate that has been brought into statistical agreement with many in situ and satellite data sets^24,36,37,61,62^. This combination of features allows ECCO4 to interpret observed thermohaline variability and attribute it to known physical mechanisms such as air-sea fluxes, advection, or diffusion^61,63–65^. Comparisons between ECCO4 and in situ Argo measurements are shown in Figs. S.3–S.4 and S.16–S.17. The close agreement between the two datasets reflects that ECCO is well constrained by Argo observations, validating the reanalysis product for the 3D Lagrangian analyses.

To delineate marine heatwaves, we use daily SST data from the Optimum Interpolation Sea Surface Temperature (OISST) product. OISST combines satellite observations, ship and buoy measurements, and employs optimum interpolation to provide globally gridded SST fields at 0.25°  spatial resolution^66–68^. OISST is commonly used for marine heatwave detection^12,15,25^. Good agreement between OISST and ECCO4 for marine heat wave metrics was recently reported in ref. ^12^ (see also Figs. S.1, S.2), which also highlights the insights provided by ECCO4’s closed heat budgets in this context^12^.

To further investigate the atmospheric forcing associated with the marine heatwave, we utilize the ERA5 reanalysis^69,70^. Combining ERA5 with OISST provides a full set of air-sea heat flux estimates (turbulent: latent and sensible; and radiative: longwave and shortwave) that can be compared with ECCO4 estimates of these quantities. ERA5 also allows us to diagnose vertically integrated atmospheric moisture and heat transports.

### Marine heatwave duration and cumulative intensity

To validate our MHW identification and assess its intensity relative to established definitions, we apply the widely-used threshold-based approach of ref. ^34^. This conventional definition facilitates comparison with existing MHW literature and confirms that the 2013–2016 event constitutes significant thermal anomalies. In this approach, an MHW is defined as a discrete period during which daily SST exceeds the seasonally varying 90th-percentile threshold of the climatological SST distribution for at least five consecutive days. The climatological baseline and percentile thresholds were computed from 1992–2017 OISST data to maintain consistency with the other analyses, which is slightly shorter than the commonly used 30-year reference window^34^. This event-based approach is widely used in MHW research and facilitates comparison between this work and the existing literature^71^. We quantified MHW severity using two complementary metrics: MHW Duration: For each grid cell and season, we calculated the total number of days classified as MHW days (i.e., days belonging to events meeting the 5-day minimum criterion). The theoretical maximum seasonal duration is 92 days (MAM and JJA each contain 92 calendar days). Cumulative Intensity: For each grid cell and season, we summed the daily intensity across all MHW days in that season, where daily intensity was defined as the excess of daily SST above the seasonal climatological mean SST^34^. This metric integrates both anomaly magnitude and event persistence into a single measure with units of ° C ⋅ days. Seasons were defined as December–February (DJF; austral summer), March–May (MAM; autumn), June–August (JJA; winter), and September–November (SON; spring). For DJF of year *n*, December of year *n* − 1 was grouped with January–February of year *n*. Duration and cumulative intensity based on OISST and ECCO are shown in Figs. 2 and S.18, respectively, and appear nearly identical.

### Temperature tendency equation in ECCO

In ECCO, temporal changes in heat content within each grid cell are explained up to machine precision by external (surface) forcing and the convergence of advective, and diffusive fluxes^36,72,73^. This forms the basis of the temperature tendency equation, which offers a physically grounded explanation for observed temperature variations. Using both the heat and volume budgets, we express the (potential) temperature tendency equation as in Eq. (1).

1 $$\frac{\partial \Theta }{\partial t}+{\mathbb{A}}(U,\Theta )={{{{\mathcal{D}}}}}_{h}(K,\Theta )+{{{{\mathcal{D}}}}}_{v}(K,\Theta )+{{{\mathcal{Q}}}}(\Theta )+{\delta }_{NA},$$

2 $$\frac{\partial \overline{\Theta }}{\partial t}+{\mathbb{A}}(U,\overline {\Theta })=\overline{{{{{\mathcal{D}}}}}_{h}(K,\Theta )}+\overline {{{{{\mathcal{D}}}}}_{v}(K,\Theta )}+\overline{{{{\mathcal{Q}}}}(\Theta )}+\overline {{\delta }_{NA}}- \overline {{\mathbb{A}}(U{\prime},\Theta ^{\prime} )}+{\mathbb{A}}(U^{\prime},\overline {\Theta }),$$

3 $$\frac{\partial \Theta ^{\prime} }{\partial t}+{\mathbb{A}}(U,\Theta ^{\prime} )=	 \underbrace{{{{{\mathcal{D}}}}}_{h}(K,\Theta )^{\prime} }_{{{{\rm{Horizontal}}}}\,{{{\rm{diffusion}}}}}+\underbrace{{{{{\mathcal{D}}}}}_{v}(K,\Theta )^{\prime} }_{{{{\rm{Vertical}}}}\,{{{\rm{diffusion}}}}}+\underbrace{{{{\mathcal{Q}}}}(\Theta )^{\prime} }_{{{{\rm{Surface}}}}\,{{{\rm{Heating}}}}}+\underbrace{\delta ^{\prime} }_{{{{\rm{NA}}}}\,{{{\rm{advection}}}}} \\ 	+\underbrace{\overline{{\mathbb{A}}(U^{\prime},\Theta ^{\prime} )}}_{{{{\rm{Rectified}}}}\,{{{\rm{advection}}}}}+\underbrace{\left(-{\mathbb{A}}(U^{\prime},\overline{\Theta })\right)}_{{{{\rm{Anomalous}}}}\,{{{\rm{advection}}}}},$$

where ${\delta }_{NA}={\mathbb{A}}(U,\Theta )-{{{\mathcal{A}}}}(U,\Theta )$ and the operators are defined as follows. First, the equation for $\frac{\partial \Theta }{\partial t}$ is obtained by combining the heat content change (*ΔH*) and volume change (*ΔV*) between time *t*_0_ and *t*_1_ according to $({t}_{1}-{t}_{0})\frac{\partial \Theta }{\partial t}=\left(\frac{\Delta H}{\rho {c}_{p}}-\Theta \Delta V\right)/{V}_{0}$, where *ρ* is the density, *c*_*p*_ is the specific heat capacity, and *V*_0_ is the volume at rest. Next, we apply the same rule to advection, diffusion, and forcing divergence terms, diagnosed exactly by MITgcm (online), and thus obtain the divergence of advective flux ${{{\mathcal{A}}}}$, the horizontal (vertical) convergence of diffusive flux ${{{{\mathcal{D}}}}}_{h}$ (${{{{\mathcal{D}}}}}_{v}$), and the external (surface) heating ${{{\mathcal{Q}}}}$. Finally, using the same advective scheme as ECCO4, we recompute the advection term offline, ${\mathbb{A}}(U,\Theta )$, using daily mean temperature and flow fields. As the advection scheme involves the product of velocity and temperature, sub-daily variation of *U* and *Θ* can be correlated, i.e. $(\hat{U\Theta })\ne \hat{U}\hat{\Theta }$, where $\hat{*}$ denotes the daily average. This is why *δ*_*NA*_ is small but not zero.

*Θ* is the (potential) temperature, *U* is the sum of Eulerian velocity and eddy bolus velocity^74^, *K* is diapycnal plus isopycnal diffusivity^75^, ${{{\mathcal{Q}}}}$ is diabatic heating (radiative and turbulent fluxes across the sea surface, plus geothermal heating at the sea floor), and *δ*_*NA*_ represents the not attributed (NA) component of advection. Since ${{{\mathcal{Q}}}}$ is concentrated at the surface, we also refer to it as surface heating.

The overlines ($\overline{*}$) in Equations (2) and (3) represent the climatological mean seasonal cycle, defined as the average of all observations for each calendar day (day-of-year) throughout the study period. To focus on temperature anomalies ($\Theta ^{\prime}=\Theta -\overline{\Theta }$), as needed to study the MHW, we calculate the anomaly between Equation (1) and its mean seasonal climatology (1992 to 2017, Equation (2)). This subtraction is essential because the mean seasonal cycle of temperature is comparable in heat content change to the temperature anomaly, as shown in Fig. S.9. As a result, we have the *temperature anomaly tendency equation* (Equation (3)). An important consideration is that Equation (3) is suitable for Lagrangian calculations because its left hand side is a valid material derivative for *U* (as opposed to $\overline{U}$ or $U{\prime}$). Also note that ${{{\mathcal{D}}}}^{\prime}$ and ${{{\mathcal{D}}}}^{\prime}$ should be interpreted as the anomalies in diffusive flux convergence—not as diffusion acting on $\Theta ^{\prime}$—because, for example, *K* varies in the mixed layer. In ECCOv4r4, the total vertical diffusivity includes contributions from the background diffusivity (optimized through the adjoint method)^36^, convective instability, the GGL90 mixed-layer parameterization^76^, and the Redi eddy-induced parameterizations^75^, resulting in spatially- and vertically-varying coefficients estimated during the model optimization. Similarly, the upward longwave component within ${{{\mathcal{Q}}}}^{\prime}$ depends non-linearly on the value of *Θ* – not just on $\Theta ^{\prime}$. We use the surface and diffusive heat flux from online model output to calculate the budget. For more detail, we refer the reader to ref. ^77^.

### Definition of MHW and passive tracer experiment

The MHW is defined as the largest contiguous volume with a temperature anomaly exceeding 1. 5 °C within the Northeastern Pacific (NEP), bounded by 20° N to 60° N and 180° W to 90° W. Nominally, we set the depth limit at 200 m, but the MHW only reaches a depth of approximately 150 m (Fig. S.8). To track the MHW, we conduct a series of passive tracer experiments using the ECCO Modeling Utilities (EMU) under no-flux boundary conditions^78^. The passive tracer is transported using the same advection and diffusion scheme as heat, but using weekly-mean flow fields and diffusivities.

At the beginning of each month in 2014 and 2015, we perform forward passive tracer experiments by initializing the tracer within the MHW region at that time with a uniform concentration of 1 mol/m^3^. Each of these 24 experiments, corresponding to different initialization times, is run until March 1, 2016, with the resulting tracer fields representing Lagrangian snapshots of the evolving MHW. We then combine the passive tracer fields by taking a maximum of all the resulting tracer concentration in each grid box. The resulting composite distribution represents the Lagrangian volume of all water that participated in the height of the MHW in 2014 and 2015. After excluding grid cells with concentrations lower than 0.1 mol m^−3^ ( < 10% of the release concentration), this tracer distribution is used to initialize the Lagrangian particle simulation described in Section 4.5.

Adjoint (backward-in-time) tracer simulations are a valuable, complementary tool for cross-validating particle trajectory calculations. Using the combined tracer distribution as the initial condition, we run the adjoint tracer simulation using EMU^78^ backward from March 1, 2016, to September 1, 2014. Comparisons between the adjoint tracer distributions and the corresponding particle locations are presented in Figs. 1i–l, S.6, S.8.

### Lagrangian budget method

Following ref. ^77^, we construct interpolated space-time fields of temperature, velocity, forcings, and flux divergences based on Equation (3). Particles are advected by the 3D velocity field $\vec{u}$, interpolated from *U*. Trajectories are computed using the Python package seaduck^79^, which implements the piecewise-stationary trajectory scheme^80^. Temperature changes experienced by the particles can be explained by the temporal integration of the interpolated right-hand-side terms of Equation (3) along particle trajectories. This approach, referred to as the Lagrangian budget, is used here to establish causal links between the temperature changes observed in the MHW and upstream processes^77^. The velocity, temperature, forcing, and divergence fields are updated daily based on the daily ECCO4 output.

In ECCO4, the effect of mesoscale eddies is represented by the GM bolus velocity, which is included in *U*, and isopycnal diffusion, which is represented by the 3D divergence of diffusive fluxes included in the heat budgets. One way of capturing the influence of mesoscale eddies on particle trajectories is to add stochastic dispersion^81^, which is a direction that we plan to explore in the future. It can be shown that the probability distribution of stochastic particles is approximately equal to passive tracer diffusion if everything else remains the same under simplifying assumptions^81^. In Fig. S.6 and S.8, we show that passive tracer concentrations and particles without stochastic dispersion have very similar spatio-temporal distributions, suggesting that including an explicit representation of the stirring by mesoscale eddies in the Lagrangian calculation can be deferred to a later study.

Based on the combined passive tracer distribution described in Section 4.4, Lagrangian particles are released on March 1, 2016, and simulated backward in time to September 1, 2013. Particle locations at selected times are shown in Fig. 1i–l, with the adjoint passive tracer distribution (see also Figs. S.6–8). A budget analysis is performed for each particle using seaduck^79^, with the contributions of individual processes summed over all particles and presented as space-time integrals following ref. ^77^.

### Proxy stream function

In Fig. 5, proxy stream functions are used to provide an intuitive representation of the circulation in the GOA and CC. For GOA, the proxy stream function is calculated by vertically integrating the meridional velocity. For CC, since the cooling is driven by vertical transport, the proxy stream function is defined in an offshore-alongshore axis and is obtained by integrating the vertical velocity along the onshore direction. In both regions, the selected velocity component corresponds to the flow crossing the mean isothermal surfaces, thus capturing the warming effect of anomalous circulation.

Figures S.10, 11 compare the integrated velocity field and the proxy stream functions. In both GOA and CC, proxy stream functions capture the main features of the overturning circulation. Taking a finite difference between two points in the vertical in the GOA will yield volume transport between those two points. Taking a finite difference in the horizontal direction in the CC will accurately estimate volume transport between two points in the vertical.

## Supplementary information


Supplementary Information
Transparent Peer Review file
Supplementary Video 1


## Data Availability

The ECCO4 reanalysis product used in this study can be downloaded at https://ecco-group.org/products-ECCO-V4r4.htm. The ERA5 dataset used in this study can be downloaded at https://cds.climate.copernicus.eu/datasets/reanalysis-era5-single-levels-monthly-means?tab=overview. The OISST product can be downloaded at https://www.ncei.noaa.gov/products/optimum-interpolation-sst.

## Code availability

All code used for data processing, calculation, and plotting in this study is openly available in the GitHub repository accessible at https://github.com/MaceKuailv/NEP_MHW_supp_code/ and permanently archived on Zenodo^82^.
