# Supplementary material for: Coupled air–sea interactions drove and sustained the 2013–2016 North Pacific marine heatwave
Source: Nat Commun. 2026 Aug 4;17:9396. doi: 10.1038/s41467-026-76096-0 (PMC13534446; doi:10.1038/s41467-026-76096-0)
Supplement: Supplementary file 1 [file 41467_2026_76096_MOESM1_ESM.pdf]

Supplementary Information for “Coupled air-sea interaction drove and sustained the 2013–2016 North Pacific Marine Heatwave"

Supplementary Methods

To ensure that the interior temperature of the MHW is well captured in ECCO, we compare the ECCO analysis with Argo profiles. We use quality controlled Argo data interpolated to fixed levels as reference and make virtual observations in ECCO4 at the same locations and times. The horizontal and temporal interpolations for the virtual observations use a nearest neighbor approach on the daily-mean ECCO4 dataset, while a piecewise linear interpolation is used in the vertical. After collecting the dataset, we mask all missing data and those that did not pass quality control in both actual and virtual observations so that the virtual observations from ECCO4 have the same sampling behavior as Argo. In both areas defined in Fig. S.9, we find all profiles within the area (51220 profiles in the north and 20508 profiles in the south) and conduct temporal bin average for each month between January 2004 (stable coverage of Argo is established) and December 2017 (end of ECCO4 period). The comparison during the MHW period is shown in Figs. S.3a,b and S.4a,b.

As much of the temperature anomaly signal is masked by the mean seasonal cycle, we calculate and remove the seasonal mean of the bin-averaged in situ and virtual data. The resulting anomalies are shown in S.3c,d and S.4c,d. We apply the same procedures to the Argo salinity measurements and the results are shown in Fig. S.16 and S.17.

Supplementary Figures

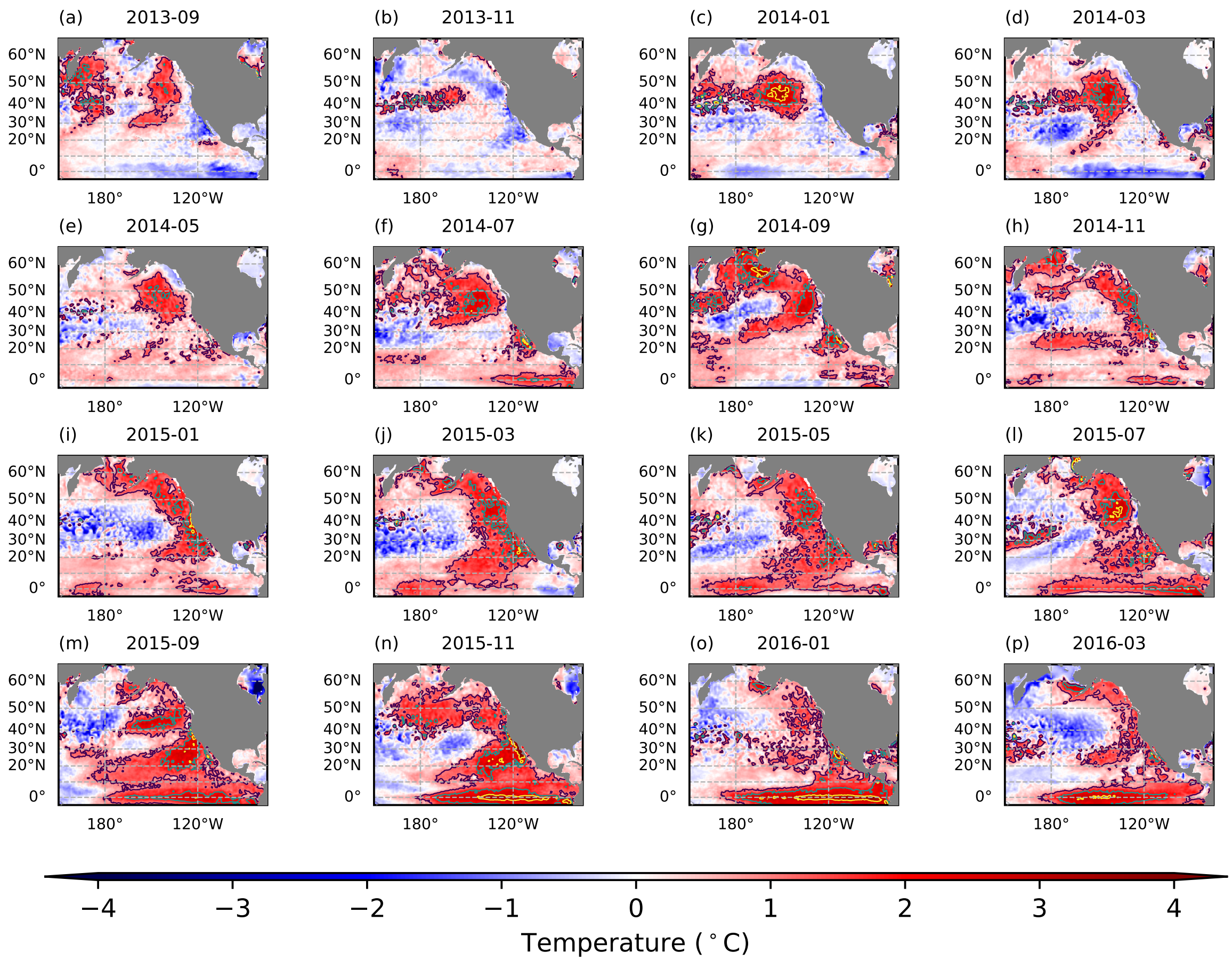

Figure S.1: Monthly mean sea surface temperature anomaly maps from the Optimum Interpolation Sea Surface Temperature product (OISST), similar to Fig. 1a–d.

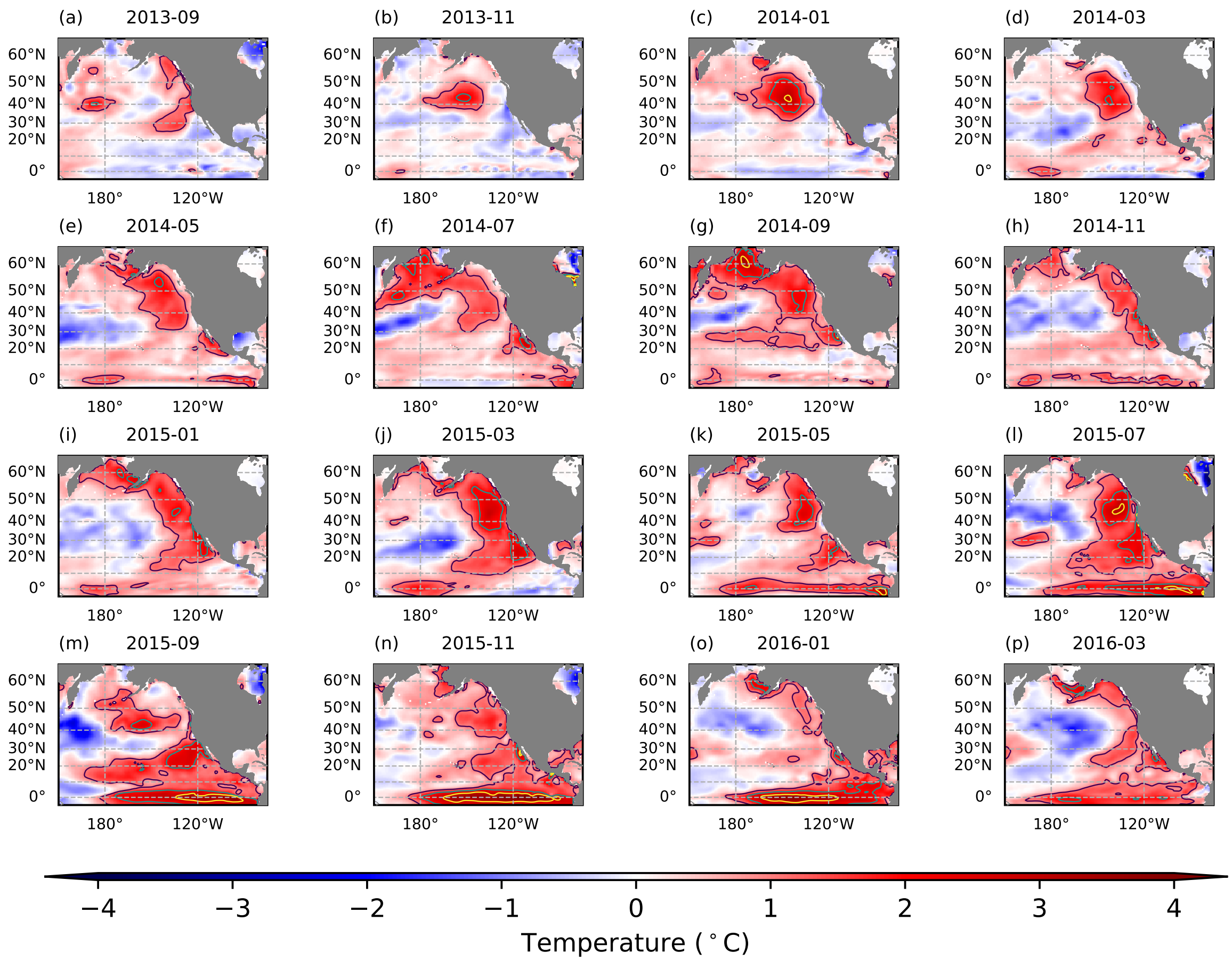

Figure S.2: Monthly mean sea surface temperature anomaly maps from the Estimating the Circulation and Climate of the Ocean, Version 4 Release 4 state estimate (ECCO4), similar to Fig. S.1

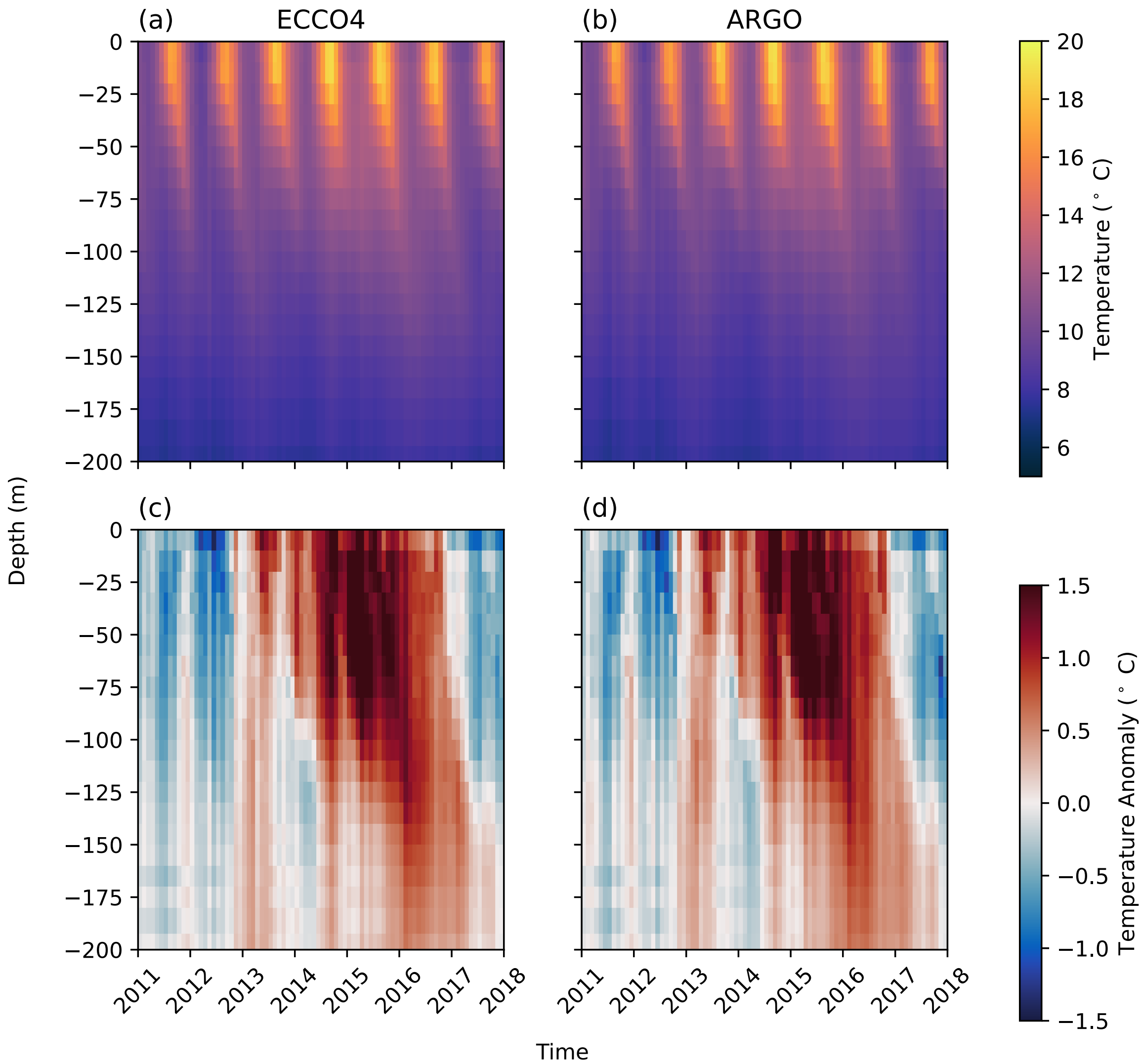

Figure S.3: Comparison between the Estimating the Circulation and Climate of the Ocean, Version 4 Release 4 state estimate (ECCO4) and Argo in situ observation in the green box in Fig. S.9a. The left column is from virtual measurements from ECCO4 (see Subsection 4.6), and the right column is from Argo in situ measurements. The top row is the temperature and the bottom row is the anomaly against seasonal mean between 2004 and 2017 (see Subsection 4.6).

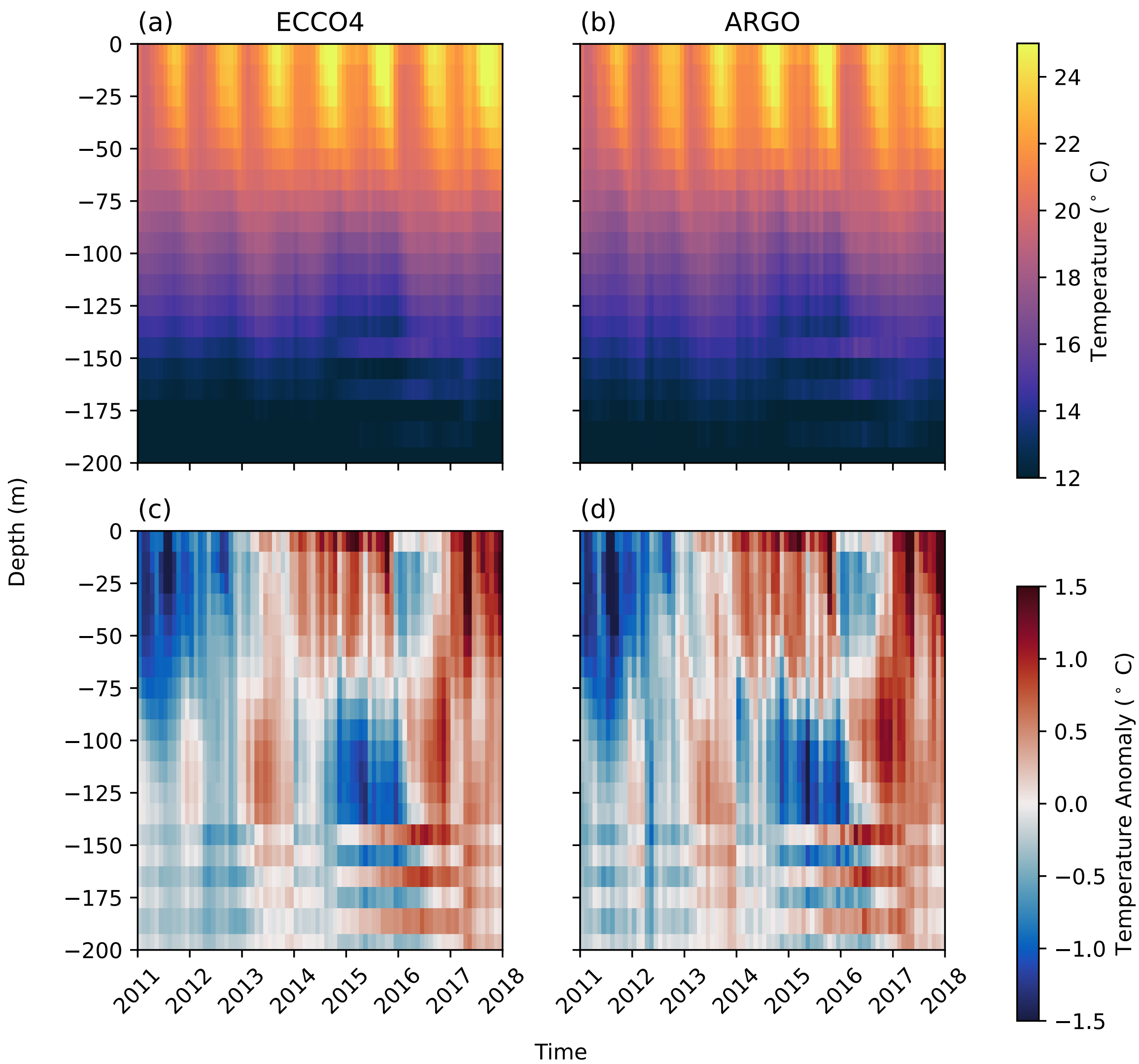

Figure S.4: Similar to Fig. S.3, but for the blue box in Fig. S.9a.

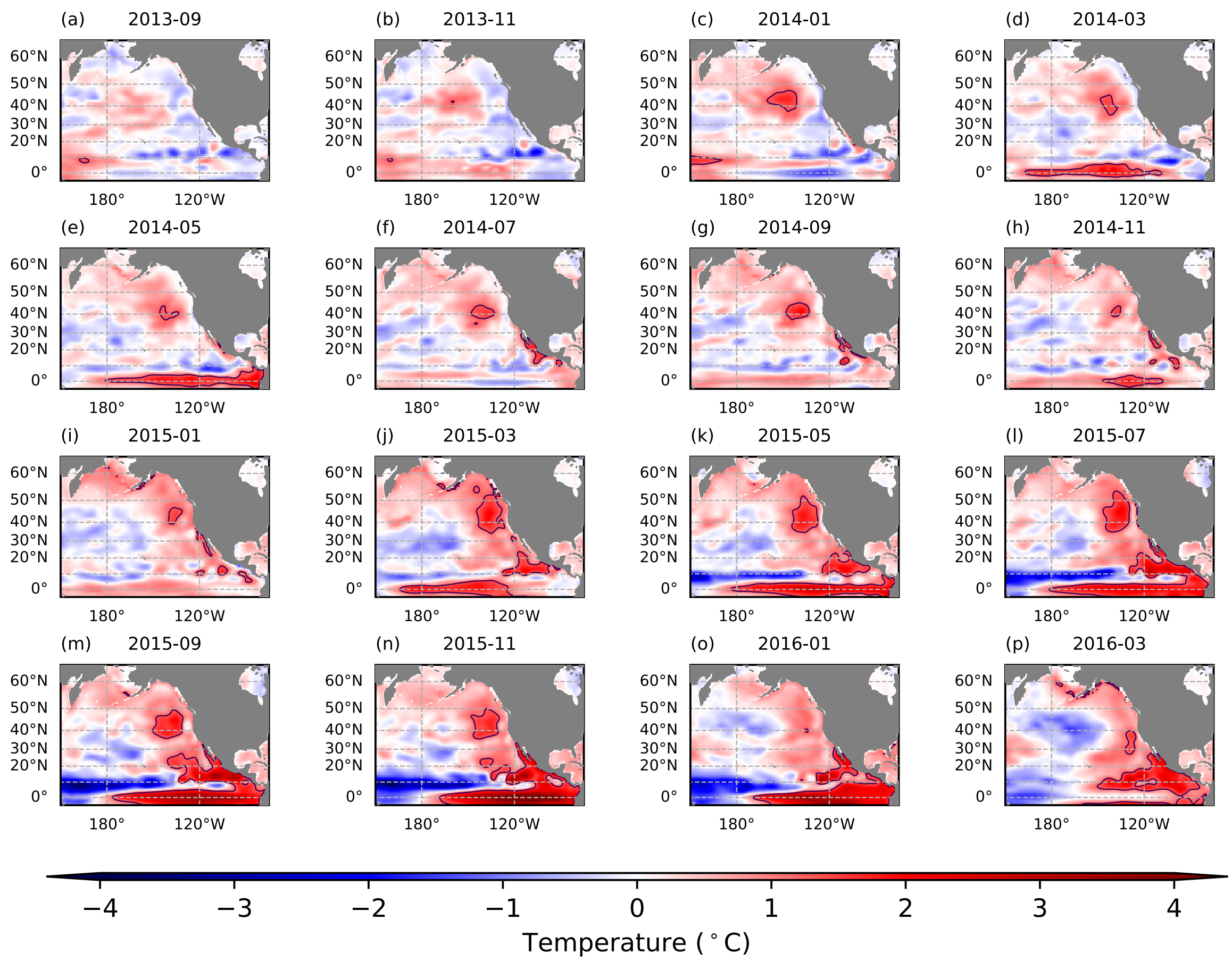

Figure S.5: Monthly mean temperature anomaly averaged in the upper 150 m from the Estimating the Circulation and Climate of the Ocean, Version 4 Release 4 state estimate (ECCO4), similar to Fig. 1e–h.

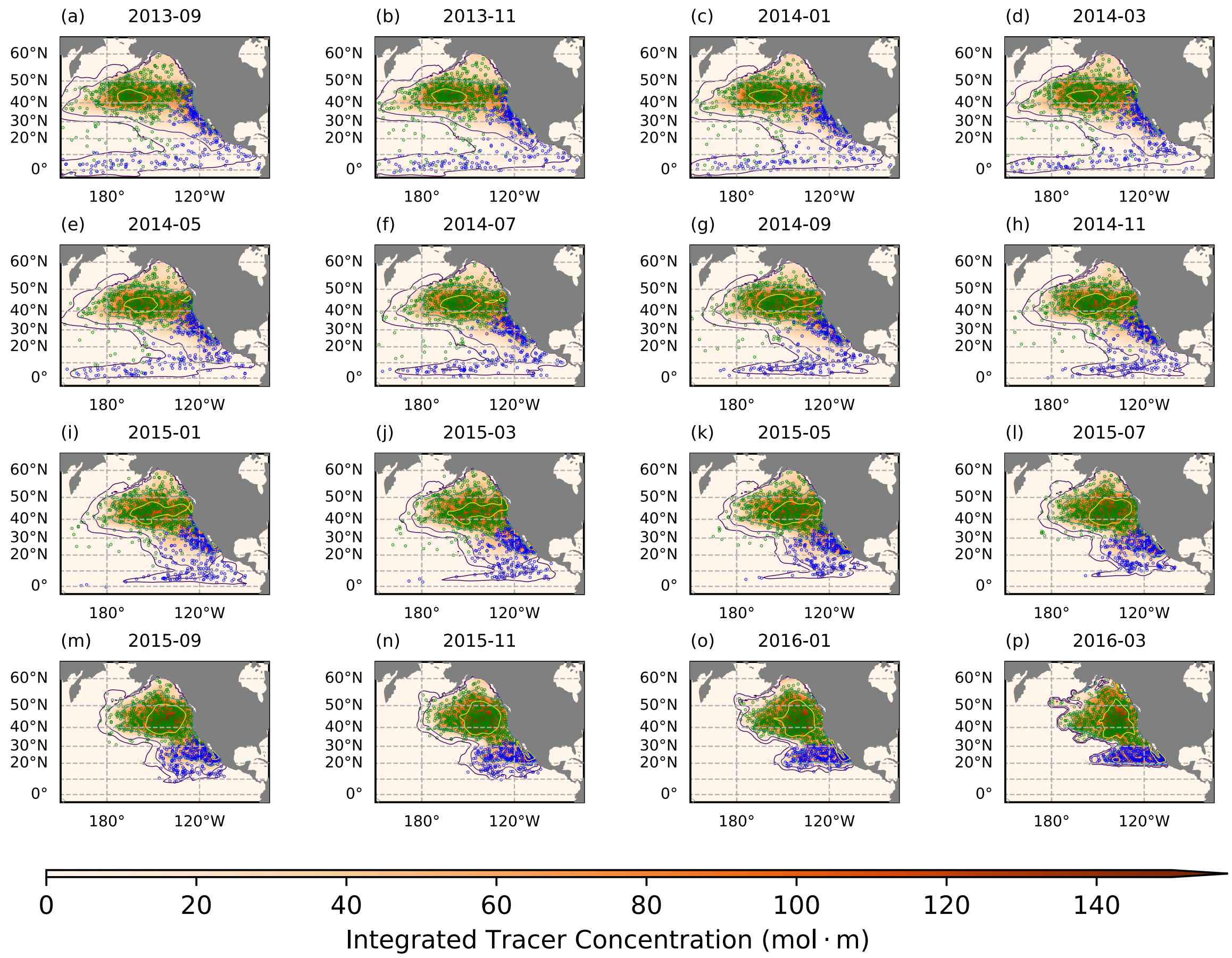

Figure S.6: Snapshots of vertical integrated passive tracer concentration and Lagrangian particle distributions, similar to Fig. 1i–l. Both tracer and particles are released in the combined marine heat wave (MHW) in March 2016, and simulated backward in time.

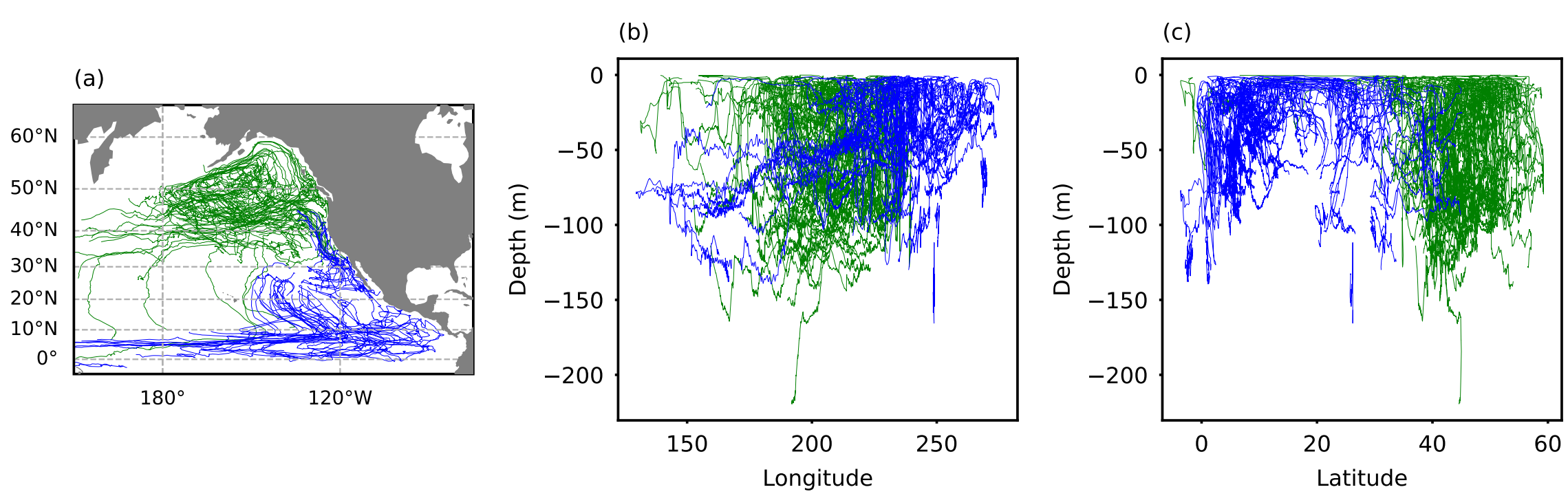

Figure S.7: Trajectories of randomly selected particles representing the northern part of the marine heat wave (N-MHW; blue, 140 particles) and the southern part of the marine heat wave (S-MHW; green, 60 particles) in the longitude-latitude (a), longitude-depth (b), and latitude-depth planes (c). Particles are released in the combined marine heat wave (MHW) in March 2016, and simulated backward to September 2014 (see Methods).

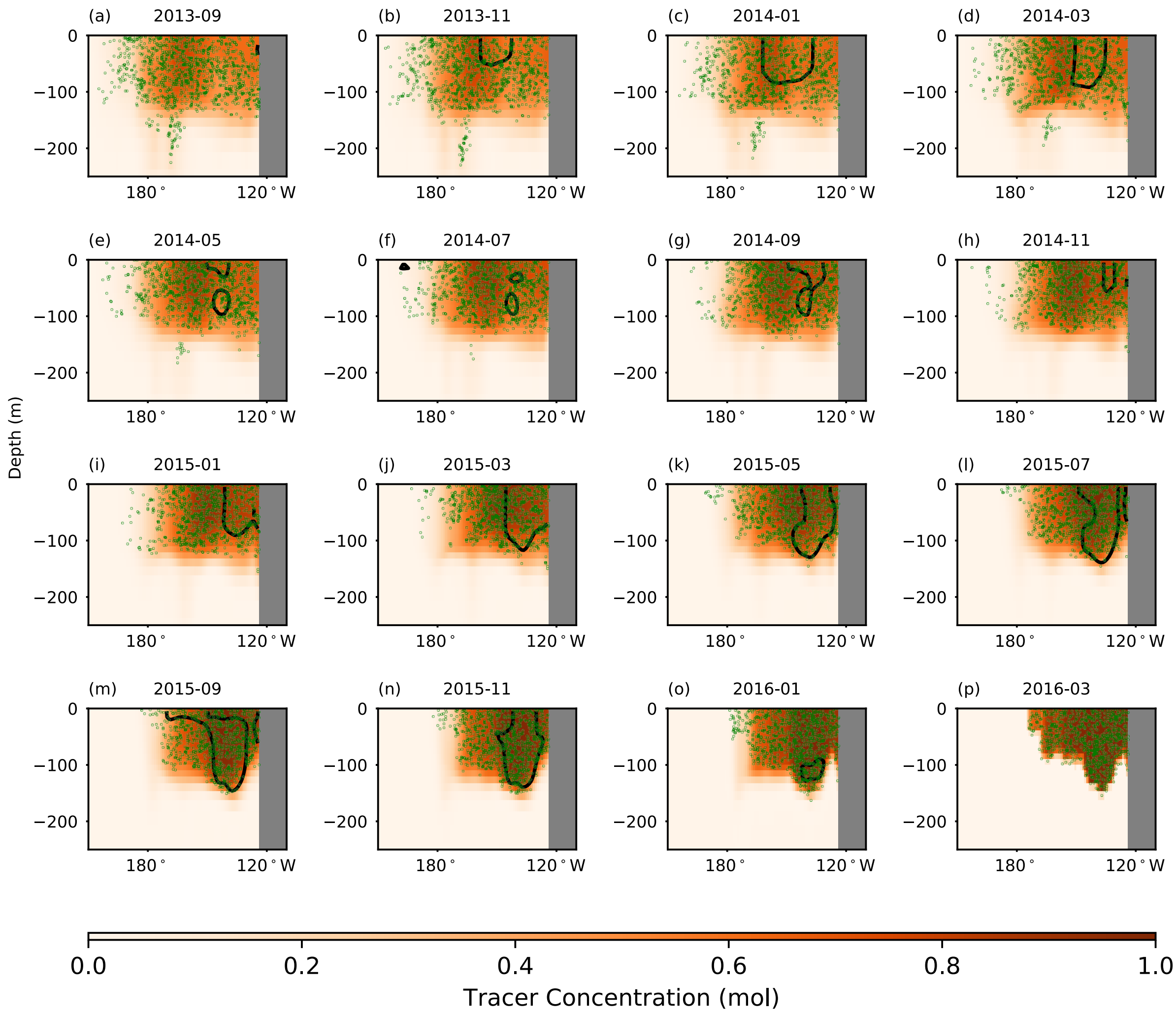

Figure S.8: Vertical sections of passive tracer concentration (shading, at 45°N), virtual particle locations (particle position between 44.9°N and 45.1°N), and the temperature anomaly (contour at 1.5°C, at 45°N).

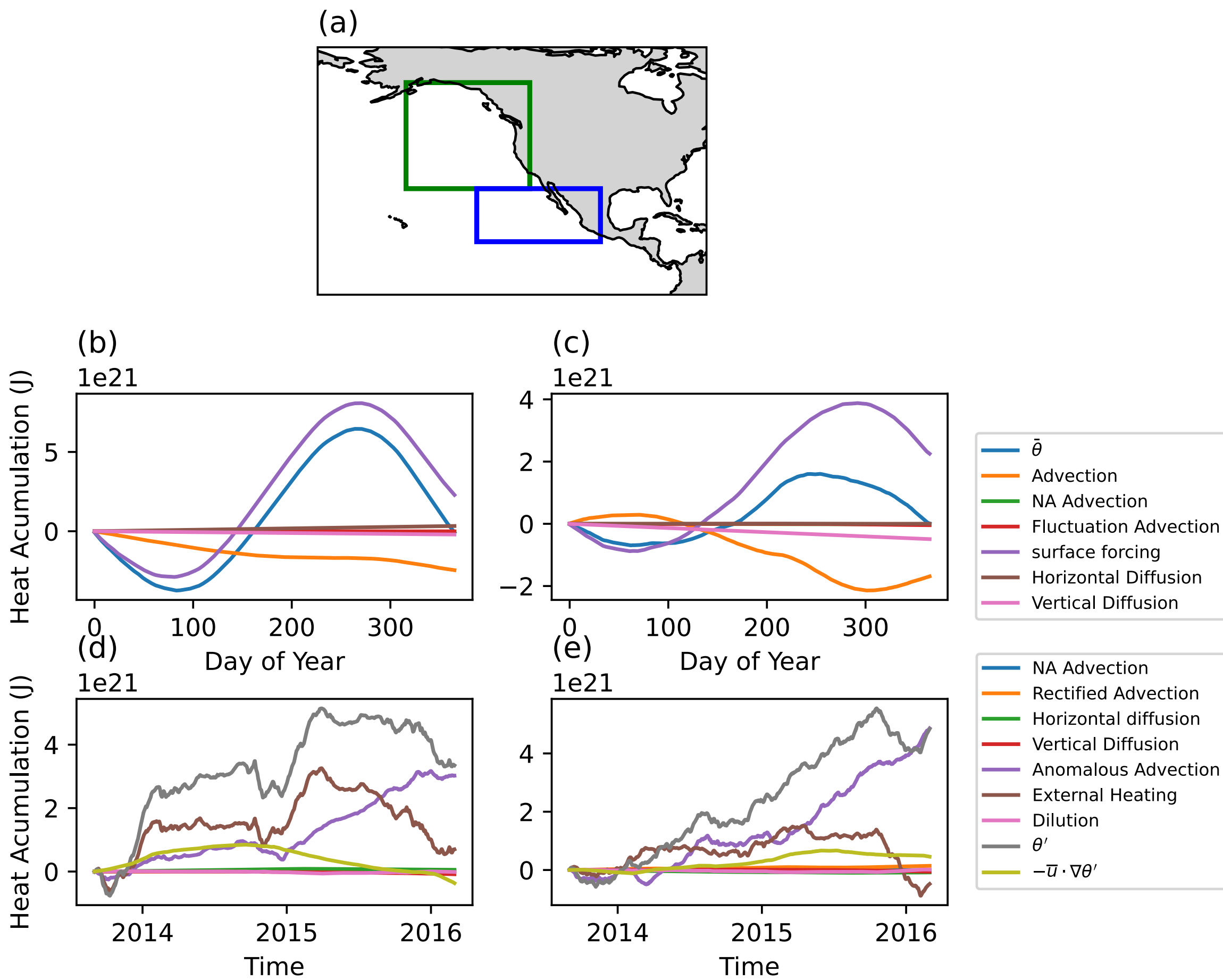

Figure S.9: (a) The areas of Eulerian heat budgets (Eqs. (2), (3)). The green box denotes the longitude-latitude region spanning 155°W to 120°W and 30°N to 60°N. The blue box covers the area from 135°W to 100°W and 15°N to 30°N. The budget results from the green (blue) box are shown in the top (bottom) row. (b) and (c) show the mean seasonality of temperature $\overline{\theta}$ and the cumulative sum of each term in the mean heat budget in the green and blue boxes, respectively. (d) and (e) are similar but show the seasonal anomalies and terms in the heat anomaly budget.

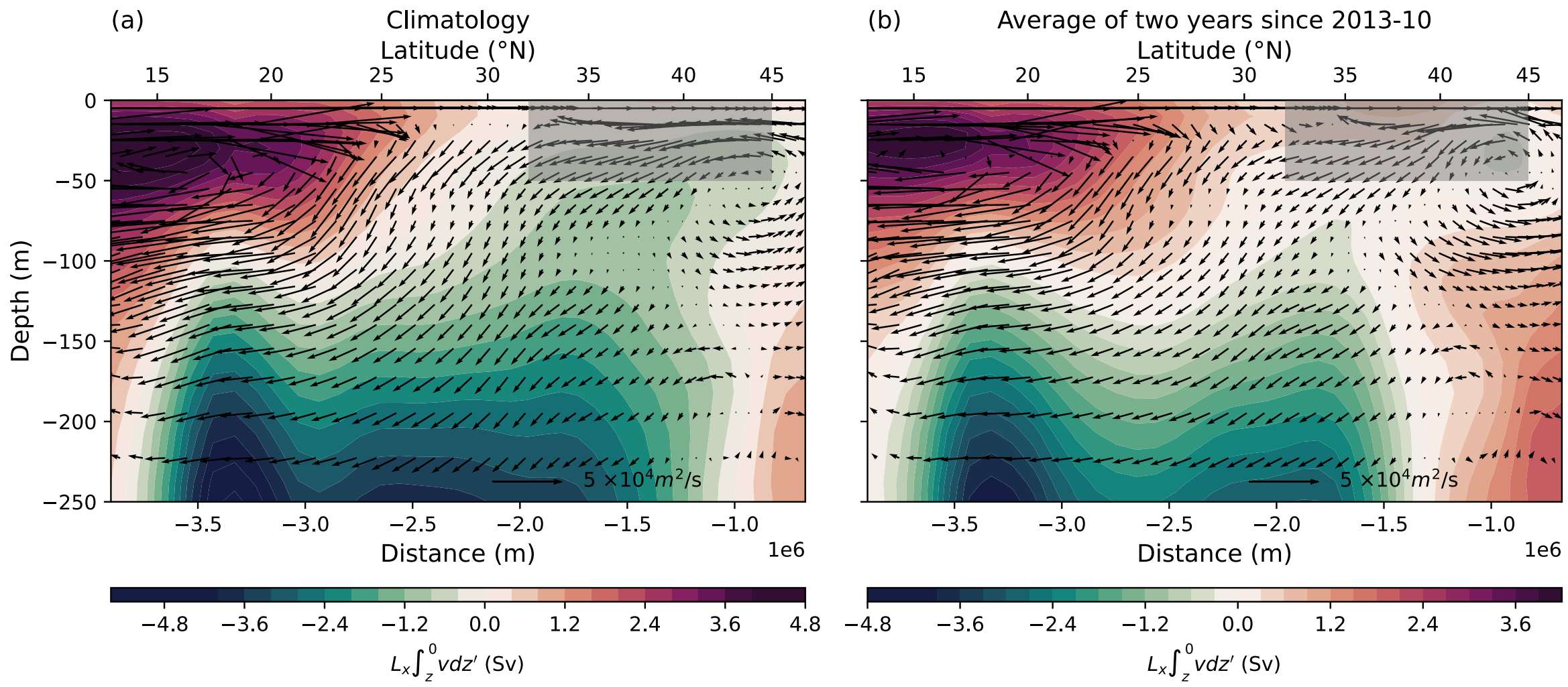

Figure S.10: Comparison of overturning representations in the Gulf of Alaska (GOA). The arrows show the zonally integrated (within the grey box in Fig. 3) meridional and vertical components of the velocity in the plane of the figure. Because of the large aspect ratio of the domain, the angles of the arrows are scaled the same way as the domain, i.e., the arrows are parallel to water parcel trajectories. The shadings are the proxy overturning stream function, as in Fig. 5a,b.

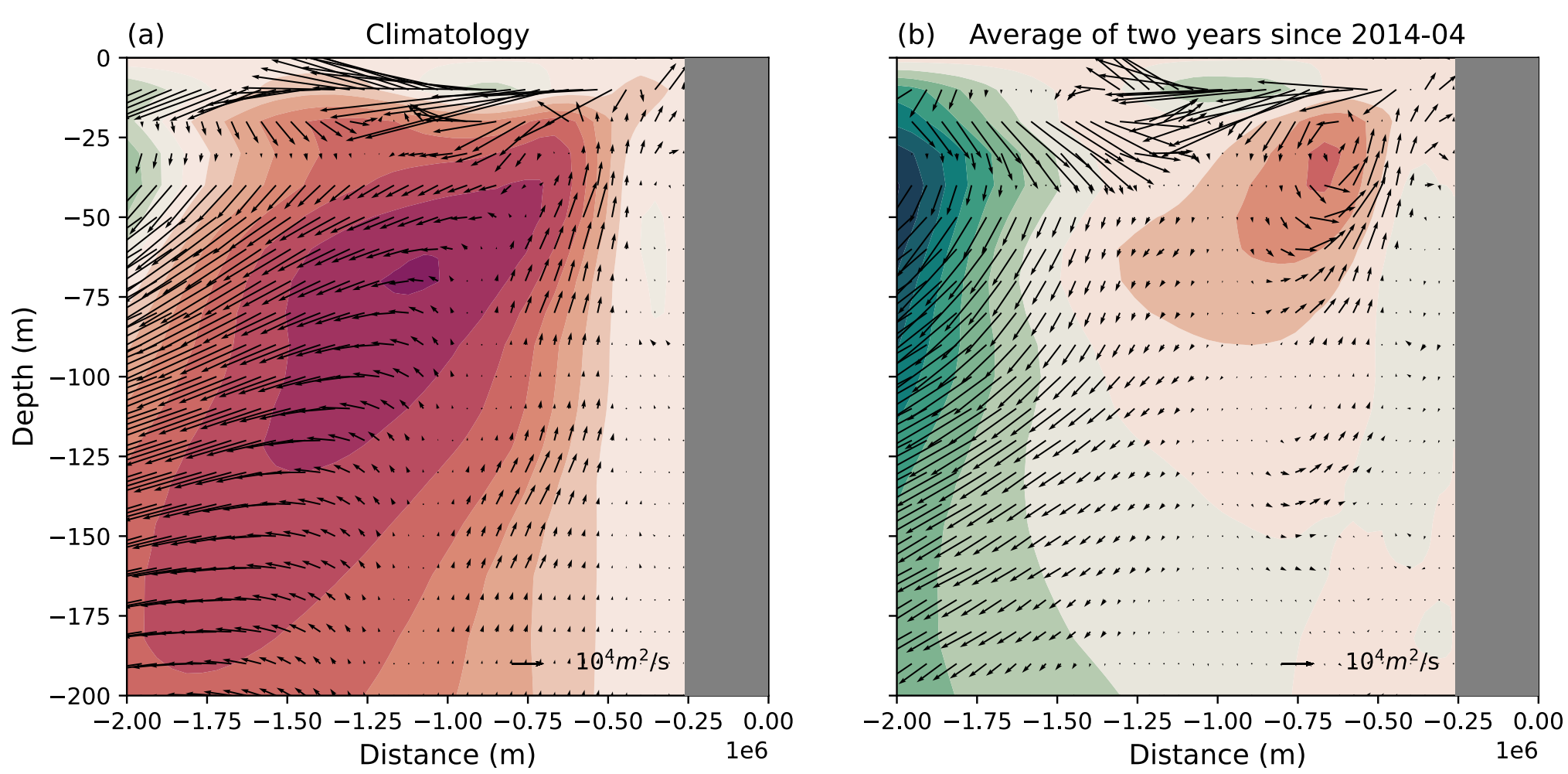

Figure S.11: As Fig. S.10, except for the California Current.

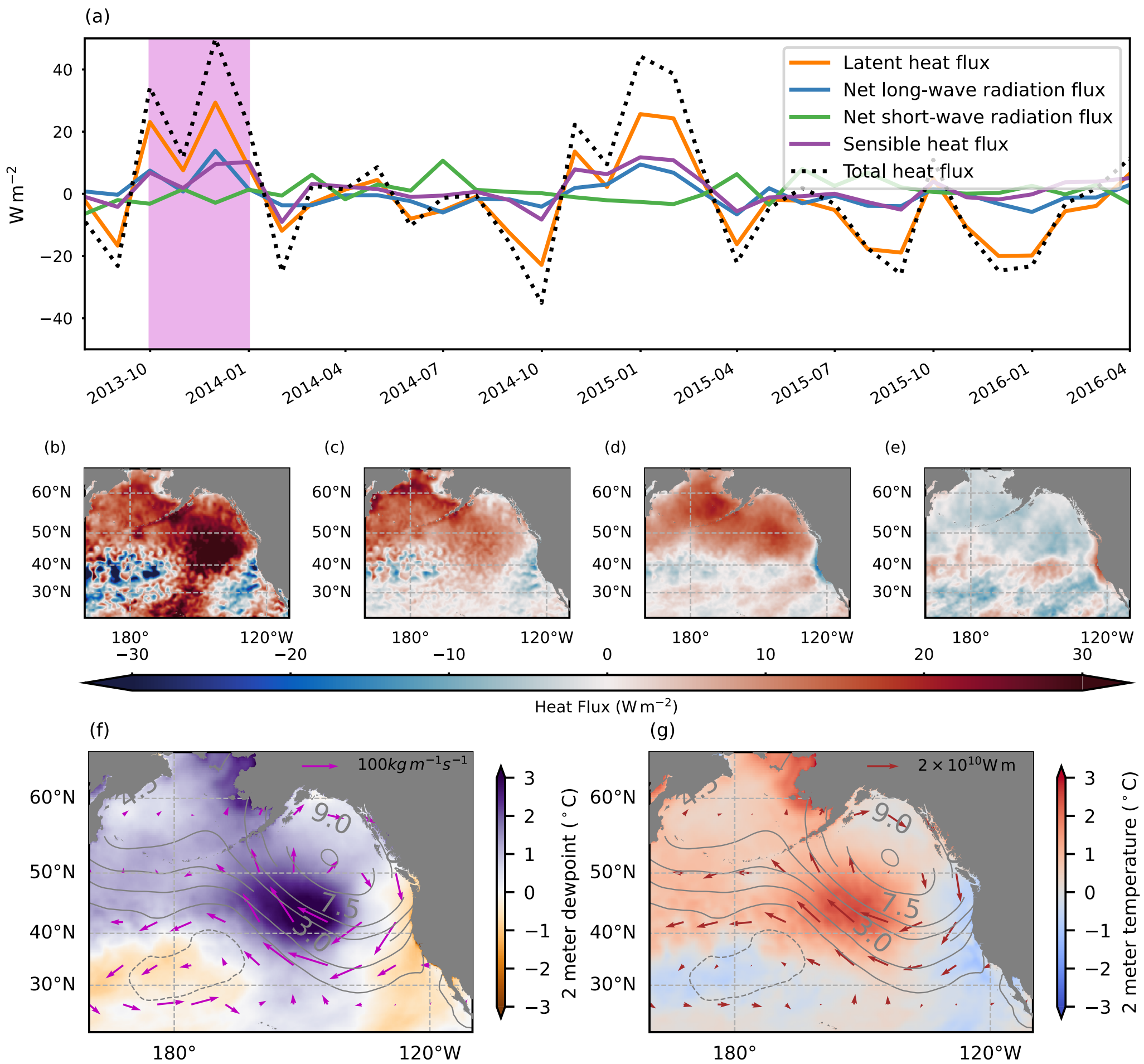

Figure S.12: Analysis of the warming from surface heat flux similar to Fig. 6, but for October, November, and December of 2013. A high sea level pressure (SLP) system near the Alaskan coast is ultimately responsible for this warming.

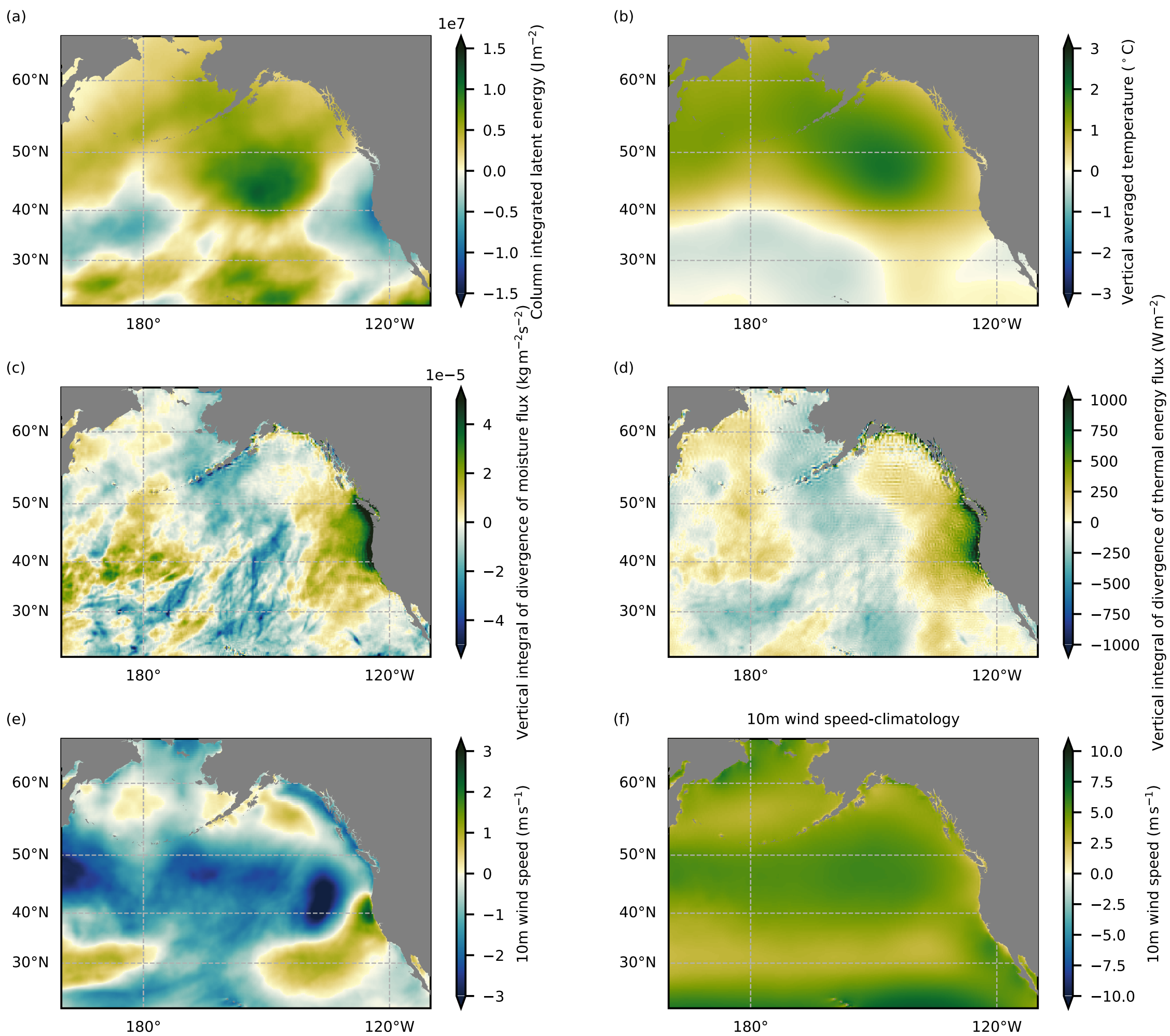

Figure S.13: Column integrated thermodynamic variables (a-d) and anomalous wind speed (e) that influence surface heat flux but are not included in Fig. S.12, similar to Fig. S.15. Panel (f) shows the climatological mean wind speed of October, November, and December.

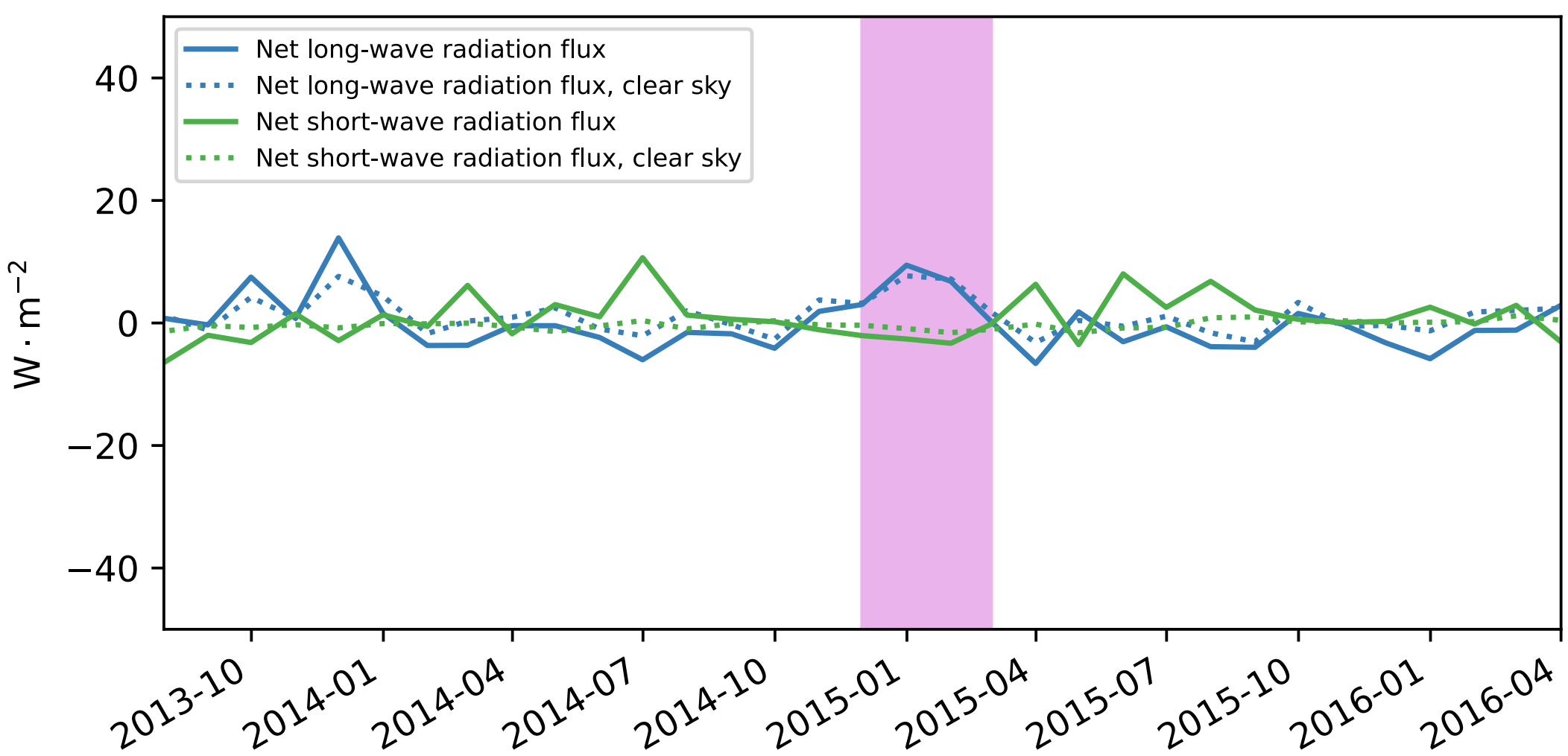

Figure S.14: Time series of radiative fluxes (solid lines, downward fluxes are positive) and the corresponding clear sky flux (dotted lines) averaged in the cyan box in Fig. 3, similar to Fig. 6a.

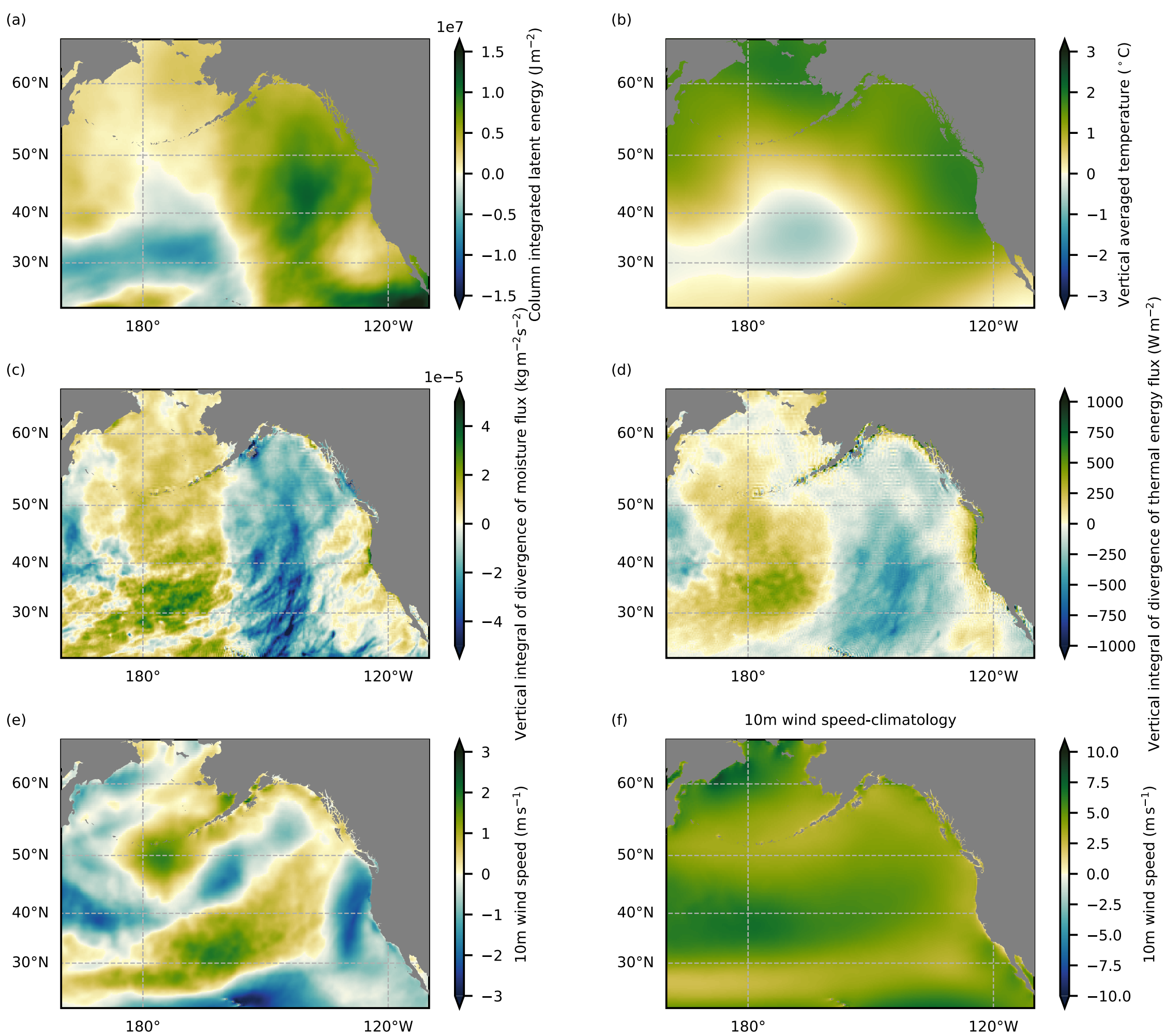

Figure S.15: Column integrated thermodynamic variables (a-d) and anomalous wind speed (e) that influence surface heat flux but are not included in Fig. 6. Negative divergences of lateral heat and moisture flux (c–d) cause increase in column integrated latent energy (a) and vertically integrated temperature (b), which help to trap longwave radiation (Fig. 6d) and are positively correlated with surface air temperature and dew point (Fig. 6f–g). Higher wind speed (e) facilitates surface turbulent heat loss (Fig. 6 b–c). For comparison, Panel (f) shows the climatological mean wind speed of December, January, and February.

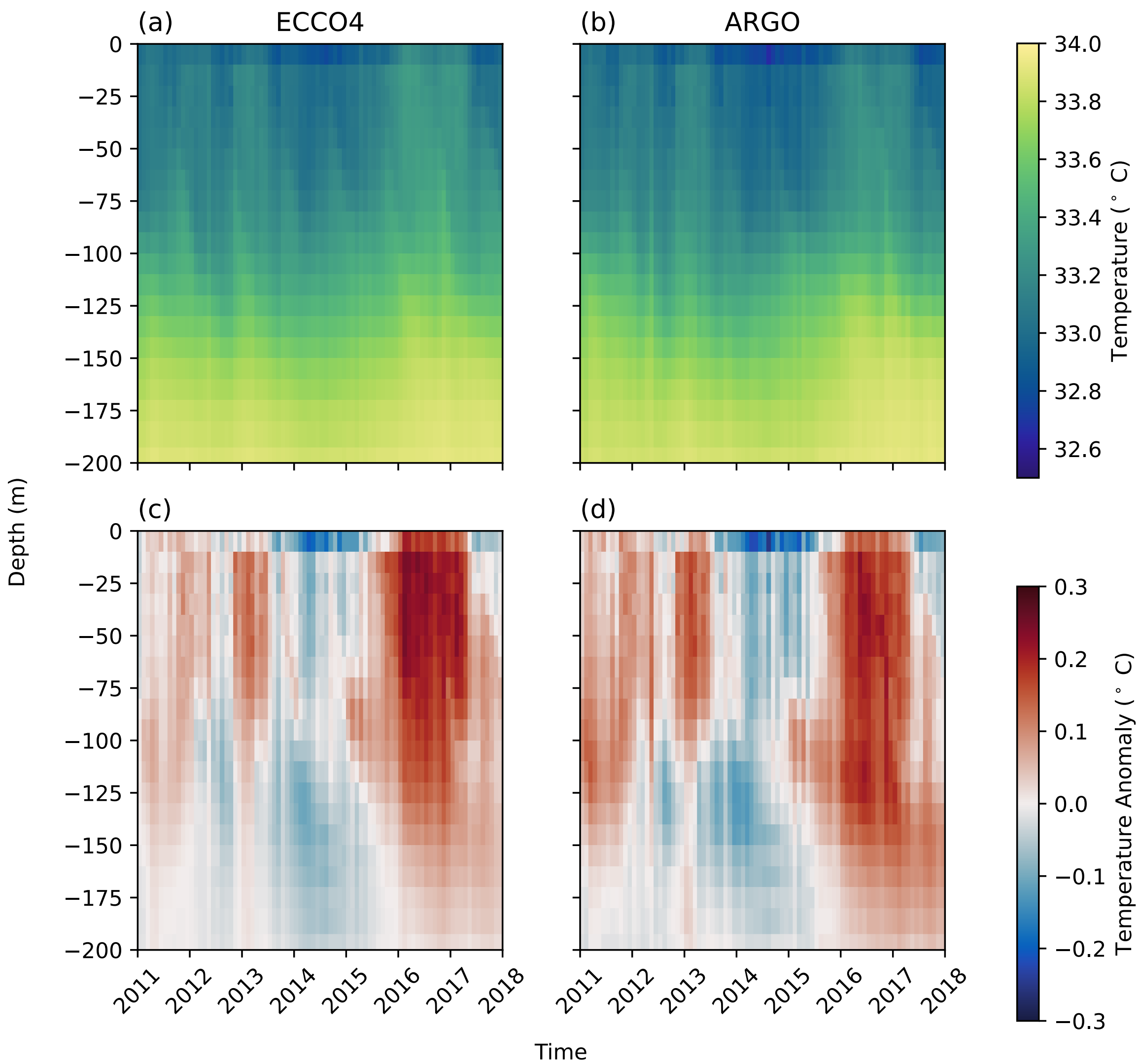

Figure S.16: Similar to Fig. S.3, but for salinity instead of temperature.

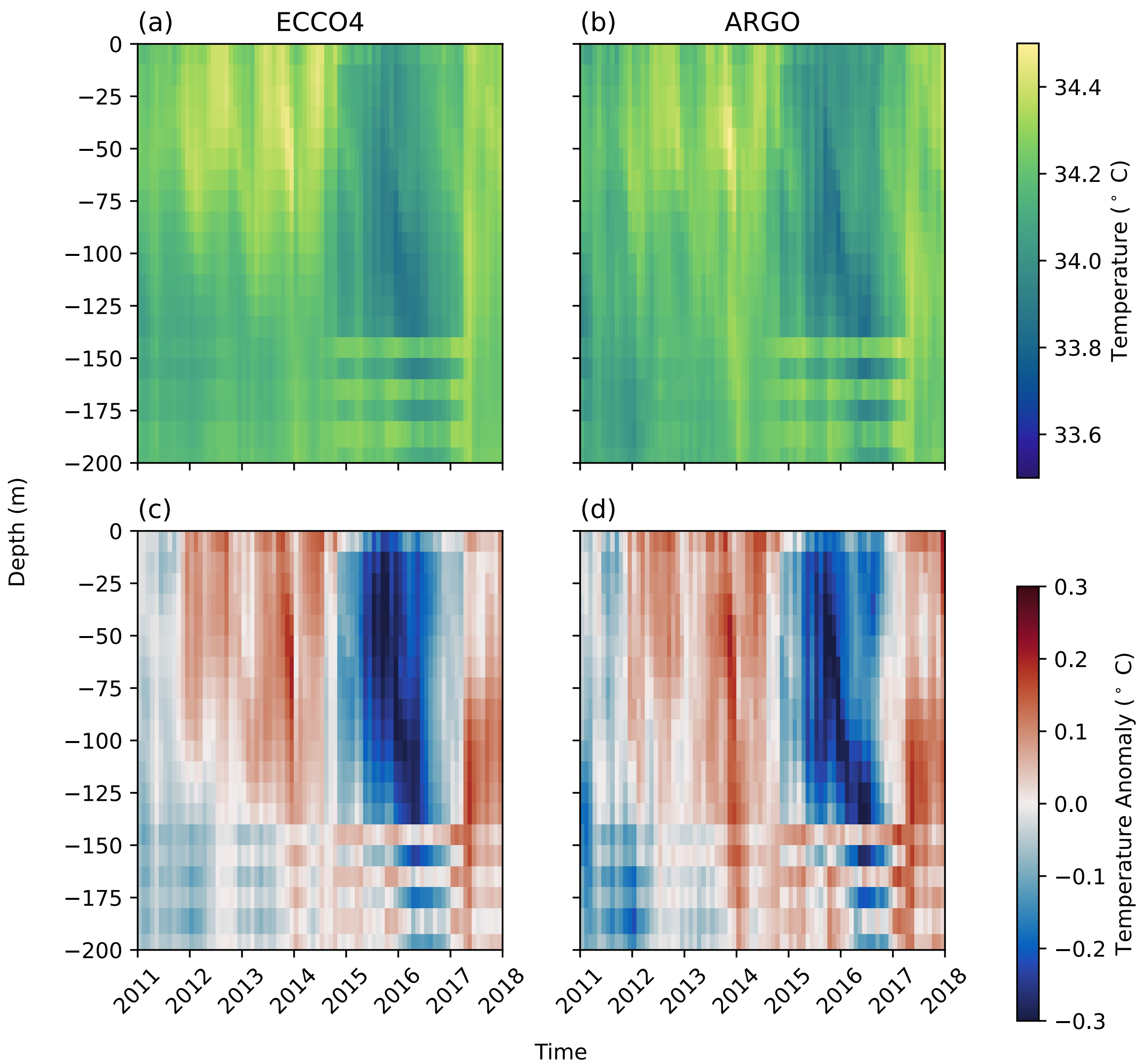

Figure S.17: Similar to Fig. S.4, but for salinity instead of temperature.

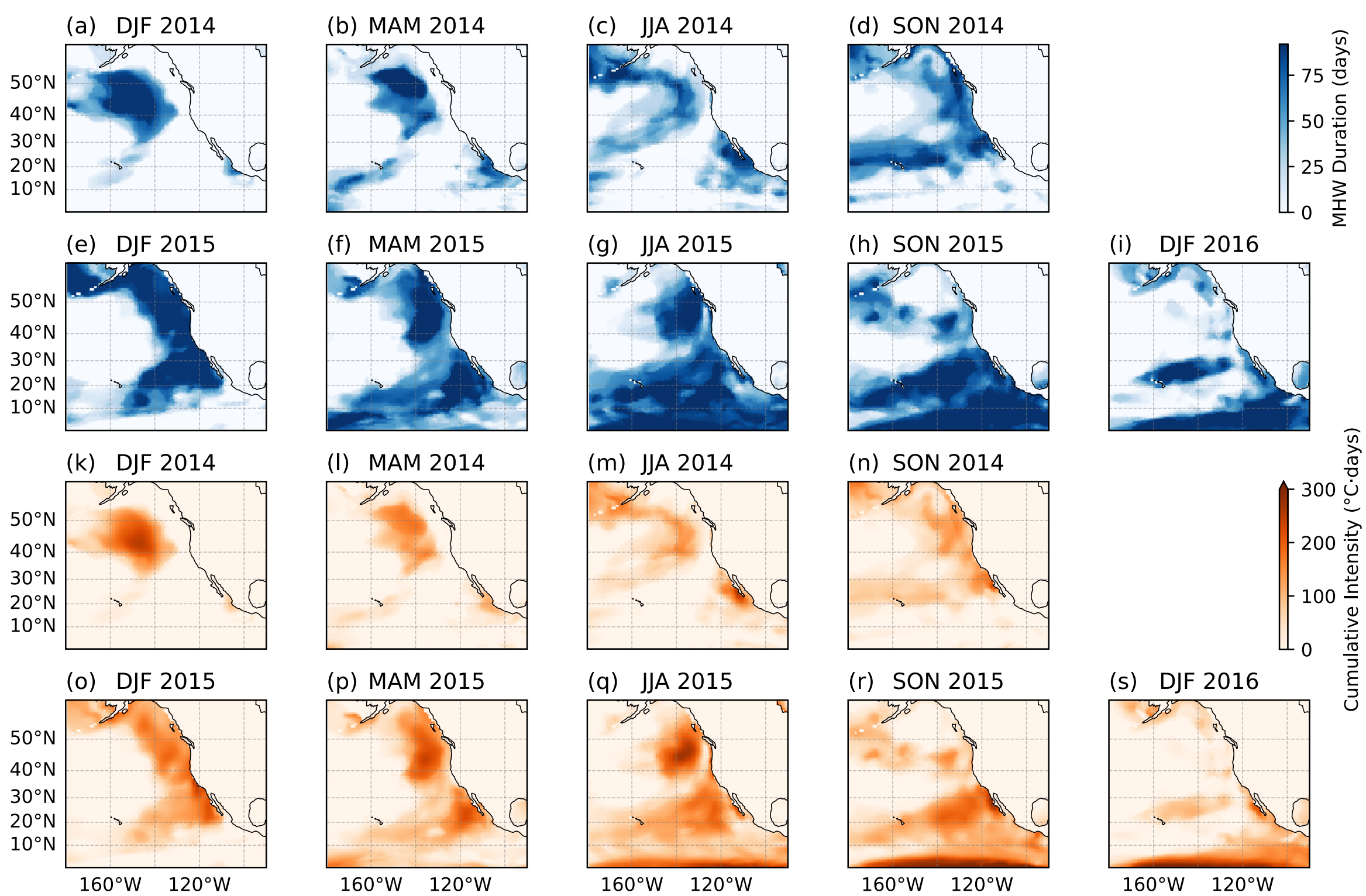

Figure S.18: Similar to Fig. 2, but based on the sea surface temperature (SST) of the Estimating the Circulation and Climate of the Ocean, Version 4 Release 4 state estimate (ECCO4).
