# Supplementary material for: Coupled air–sea interactions drove and sustained the 2013–2016 North Pacific marine heatwave
Source: Nat Commun. 2026 Aug 4;17:9396. doi: 10.1038/s41467-026-76096-0 (PMC13534446; doi:10.1038/s41467-026-76096-0)
Supplement: Supplementary file 2 [file 41467_2026_76096_MOESM2_ESM.pdf]

nature portfolio

Peer Review File

Coupled air-sea interaction drove and sustained the 2013-2016 North Pacific Marine Heatwave

Corresponding Author: Dr Wenrui Jiang

This file contains all reviewer reports in order by version, followed by all author rebuttals in order by version.

Version 0:

Reviewer comments:

Reviewer #1

(Remarks to the Author)
This manuscript investigates the mechanisms underlying the formation and persistence of the 2013–2016 marine heatwave (MHW) in the Northeast Pacific. Heat advection is analyzed using passive tracers and a novel Lagrangian heat budget approach. The study also examines the relationship between atmosphere–ocean heat exchanges and sea level pressure anomalies. The results are compelling, the analysis is thorough and generally convincing, and the discussion offers thoughtful interpretation.

Although I have several methodological concerns, I believe these can be addressed through clarification and may not require additional calculations. I recommend publication after a revision.

1) The ECCO product does not resolve mesoscale currents ("eddies"), and the trajectories of Lagrangian particles are not influenced by these eddies. Could the authors clarify how stirring and dispersal of Lagrangian particles by mesoscale eddies are accounted for in this part of the analysis? According to Section 4.4, temperature and tracer evolution are diagnosed using Equation 3, which includes horizontal diffusion intended to parameterize mesoscale eddies. However, my concern is that the locations of particles are not affected by mesoscale dynamics.

2) I think the description of methods at the bottom of page 6 needs further clarification. Why is the divergence of the advective flux different from its offline counterpart? In other words, why is the "delta-term" not zero, and how large is this term?

3) How is the climatological cycle defined? Depending on the definition, Equations 2–3 may include additional terms. For example, if climatology is defined by filtering or smoothing in time, applying a bar operator to anomalies does not necessarily yield zero.

4) Vertically integrated meridional velocity is not, strictly speaking, a streamfunction, since zonal exchanges are not accounted for. Its contour lines do not correspond to overturning circulation unless the velocity is also integrated zonally from coast to coast. Please clarify the meaning of the "proxy streamfunction" in Section 4.5. I am concerned that if the streamfunction is not properly calculated, the discussion in Section 2.2 may be misleading. Why not present the two-dimensional Ekman transport and its convergence instead?

5) Do surface winds explicitly depend on SST anomalies in this reanalysis? Could an increase in wind speed over warm SST anomalies, as described in the Vertical Mixing Mechanism (Wallace et al., 1989), be a significant factor in the lifecycle of the MHW?

(Remarks on code availability)

Reviewer #2

(Remarks to the Author)
Comments to the Nature Communications manuscript entitled "Coupled air-sea interaction drove and sustained the 2013–2016 North Pacific Marine Heatwave (NCOMMS-25-85679-T)" by Jiang et al.
General Comments
The authors investigate the mechanisms responsible for the anomalously prolonged marine heatwave (MHW) conditions in the northeastern Pacific Ocean during 2013–2016 using reanalysis products and OI-SST data. Given the strong influence of MHWs on marine ecosystems and their feedbacks to the overlying atmosphere, this study addresses a problem of considerable scientific and societal relevance. While the manuscript provides a coherent large-scale interpretation of the coupled air–sea processes sustaining the event, it does not sufficiently explore the seasonal evolution of the MHW. A more detailed examination of how the dominant physical processes vary across different seasons, such as winter versus summer contributions of surface heat fluxes, ocean heat transport, and mixed-layer processes, would substantially strengthen the present analysis. Therefore, I recommend that the manuscript be revised to include a more in-depth, seasonally resolved analysis of the MHW evolution, as mentioned in the comments below. With these revisions, the manuscript would be suitable for publication in Nature Communications. Hence, recommended for minor revisions before accepted for publication.
Other comments.
1) The authors mentioned that the ECCO data and OI-SST show good agreement in reproducing the MHW. However, it is unclear whether the performance of the ECCO model simulation with OISST in the NEP during the 2013-2016 study period. Hence, it is suggested to make a comparison between OISSST and ECCO to demonstrate what we saw in the MHW conditions in the OISST is rightly reproduced in the ECCO data. Similarly, readers would like to know how well the simulation of ECCO reproduces the sub-surface information up to 150 m. Hence, a comparison with Argo floats in the NEP during 2013-16 is imperative.
2) In the Results section. The northern anomaly and the southern anomalies. Please mark this area in the figure and specify this domain in the text.
3) In the Results section. The movement of the particle is mainly due to horizontal circulation. Hence, how can we connect that with marine heat wave propagation? I think a simple Hovmoller diagram of MHW intensity will provide insight into the spatial propagation of MHWs. Moreover, the spatial propagation or its westward extension can be associated with atmospheric flux impact.
4) In the Results section. Though the authors provided SST anomalies, there is no information on whether these SST anomalies are classified as MHW. It is strongly recommended to prepare a figure for MHW for SST and OHC similar to Figure E1/E3. In addition, it is also recommended to add cumulative MHW intensity in each season.
5) 2014-12 and 2015-04, the SST anomaly cannot be considered as northern and southern MHW. Since it is apparent that a warm anomaly persists through the East Coast. Even in 2015-11, the MHW is clearly a single entity.
6) The Lagrangian-based framework adopted by the authors may be difficult to follow for a broad readership. To facilitate clearer interpretation, I suggest additionally performing a simplified heat budget for the upper 150 m of the water column and comparing the individual budget terms with their climatological counterparts. Such an analysis would help clarify which physical processes primarily contribute to the maintenance of the marine heatwave in each of the two boxes.
7) In section 4.2, it is mentioned that K is the diapycnal and horizontal diffusivity. There is no information on the value of these constants that we used in the budget. Its values will be different both at the surface and the thermocline for vertical. How authros derived these informations.
8) Section 2.1. Over time scale of few months. Which months. Please specifiy.
9) Section 2.2. later 2014. The cumulative heat flux shows large heat loss. From Figure 2a.
10) I am not sure whether the authors performed the budget with respect to the 150 m or the mixed layer. If it is the first case, the heat flux impact is mainly restricted to the upper 30-40 m of the water column. Please specify.
11) Figure 2b and 2c; what is the justification for selecting a different period for constructing mean picture.

(Remarks on code availability)

Reviewer #3

(Remarks to the Author)
Review on "Coupled air-sea Interaction drove and sustained the 2013-2016 North Pacific Marine Heatwave" by Jiang et al.

This study investigates the 2013–2016 marine heatwave (MHW) events in the northeastern Pacific Ocean. The authors employ a Lagrangian heat budget framework to track the evolution of these events and diagnose the dominant physical processes governing their development and persistence. The analysis is based primarily on the ECCO v4 release 4 global ocean state estimate. A series of passive tracer experiments are conducted using the ECCO Modeling Utilities (EMU) under no-flux boundary conditions. Observational and atmospheric reanalysis products, including OISST and ERA5, are used to qualitatively support several aspects of the results. The study highlights the roles of Ekman heat transport and Ekman upwelling in shaping the SST anomalies and further links the events to large-scale climate modes such as the Pacific Decadal Oscillation (PDO) and variability associated with the Aleutian Low. These mechanisms are summarized in a clear and well-designed schematic diagram.

Despite these strengths, I have two major concerns regarding the novelty and methodological suitability of the study.

(i) The 2013–2016 northeastern Pacific marine heatwave has been extensively documented in the literature, with numerous studies already describing its physical drivers, air–sea coupling, and large-scale climate connections. While the present

study applies the ECCO framework and a Lagrangian heat budget approach, the resulting physical interpretation largely reiterates mechanisms that are already well established, including reduced heat loss, anomalous Ekman transport, and basin-scale atmospheric forcing. The manuscript does not clearly articulate what fundamentally new insight is gained beyond re-diagnosing known processes using a different modeling framework. In addition, it is surprising that the study does not explicitly reference the term "California Blob," which has been widely used in prior literature to describe the 2013–2016 event and provides important historical and conceptual context. The absence of this terminology, without justification, further obscures the connection between this work and the existing body of research.

(ii) A more serious concern relates to the suitability of ECCO for studying marine heatwaves as they are formally defined. Marine heatwaves are typically identified as periods during which SST exceeds the 90th percentile of a climatological baseline for at least five consecutive days, following the definition of Hobday et al. (2016). ECCO state estimates are optimized to provide dynamically consistent, global ocean reconstructions that are most reliable at monthly to longer timescales. As such, ECCO is not specifically designed to resolve short-duration extreme events at the temporal precision required for MHW detection and characterization. This limitation raises questions about whether ECCO can robustly capture the onset, peak intensity, and duration of marine heatwaves as defined by percentile-based, daily SST thresholds. While ECCO may be useful for diagnosing large-scale heat transport and low-frequency variability, its application to event-scale marine heatwave analysis requires stronger justification and careful discussion of uncertainties.

Overall, while the study is clearly written and methodologically thorough within the ECCO framework, the lack of clear novelty and the potential mismatch between the chosen model product and the temporal definition of marine heatwaves substantially weaken the impact of the manuscript. Addressing these concerns, by clarifying the unique contributions of the study and more rigorously justifying the use of ECCO for MHW analysis, would be essential before the work can be considered for publication.

(Remarks on code availability)

Version 1:

Reviewer comments:

Reviewer #1

(Remarks to the Author)
The authors have adequately addressed my previous suggestions, and in my view the manuscript is now suitable for publication.

Sincerely,
Igor Kamenkovich

(Remarks on code availability)

Reviewer #3

(Remarks to the Author)
The authors have adequately addressed all reviewer comments and incorporated the suggested revisions. I believe the manuscript is suitable for publication in its present form.

(Remarks on code availability)

Open Access This Peer Review File is licensed under a Creative Commons Attribution 4.0 International License, which permits use, sharing, adaptation, distribution and reproduction in any medium or format, as long as you give appropriate credit to the original author(s) and the source, provide a link to the Creative Commons license, and indicate if changes were made.
In cases where reviewers are anonymous, credit should be given to 'Anonymous Referee' and the source.
The images or other third party material in this Peer Review File are included in the article's Creative Commons license, unless indicated otherwise in a credit line to the material. If material is not included in the article's Creative Commons license and your intended use is not permitted by statutory regulation or exceeds the permitted use, you will need to obtain permission directly from the copyright holder.
To view a copy of this license, visit https://creativecommons.org/licenses/by/4.0/

Response to Reviewers

Manuscript title: **Coupled air-sea interaction drove and sustained the 2013–2016 North Pacific Marine Heatwave**

Manuscript number: **[NCOMMS-25-85679-T]**

Reviewer #1

This manuscript investigates the mechanisms underlying the formation and persistence of the 2013–2016 marine heatwave (MHW) in the Northeast Pacific. Heat advection is analyzed using passive tracers and a novel Lagrangian heat budget approach. The study also examines the relationship between atmosphere–ocean heat exchanges and sea level pressure anomalies. The results are compelling, the analysis is thorough and generally convincing, and the discussion offers thoughtful interpretation.

Although I have several methodological concerns, I believe these can be addressed through clarification and may not require additional calculations. I recommend publication after a revision.

1) The ECCO product does not resolve mesoscale currents ("eddies"), and the trajectories of Lagrangian particles are not influenced by these eddies. Could the authors clarify how stirring and dispersal of Lagrangian particles by mesoscale eddies are accounted for in this part of the analysis? According to section 4.4, temperature and tracer evolution are diagnosed using Equation 3, which includes horizontal diffusion intended to parameterize mesoscale eddies. However, my concern is that the locations of particles are not affected by mesoscale dynamics.

The influence of mesoscale eddies are parameterized in the ECCO product. The way their influence appears in our analysis is through the GM-Redi parameterization (Gent and McWilliams 1990, Redi 1982). The trajectory of Lagrangian particles are calculated using the explicit velocity plus the GM bolus velocity. The isopycnal component of the diffusion (including both horizontal and vertical components) does not influence the particles' trajectories, but it is still accounted for in the budget. While we do not attempt to represent the dispersion by mesoscale eddies in the Lagrangian case, we carry out passive tracer calculations that include the Eulerian expression of this term via the Redi isopyncal diffusion, which is equivalent to simulating particles with stochastic dispersion (van Sebille et al., 2018). Indeed, results show that the passive tracer and particle distributions are very similar and, thus, the diffusive effect must be relatively weak. This is likely because the volume we considered is quite large, and the time scale is relatively short.

We have added a paragraph in section 4.5 to further discuss this.

2) I think the description of methods at the bottom of page 6 needs further clarification. Why is the divergence of the advective flux different from its offline counterpart? In other words, why is the "delta-term" not zero, and how large is this term?

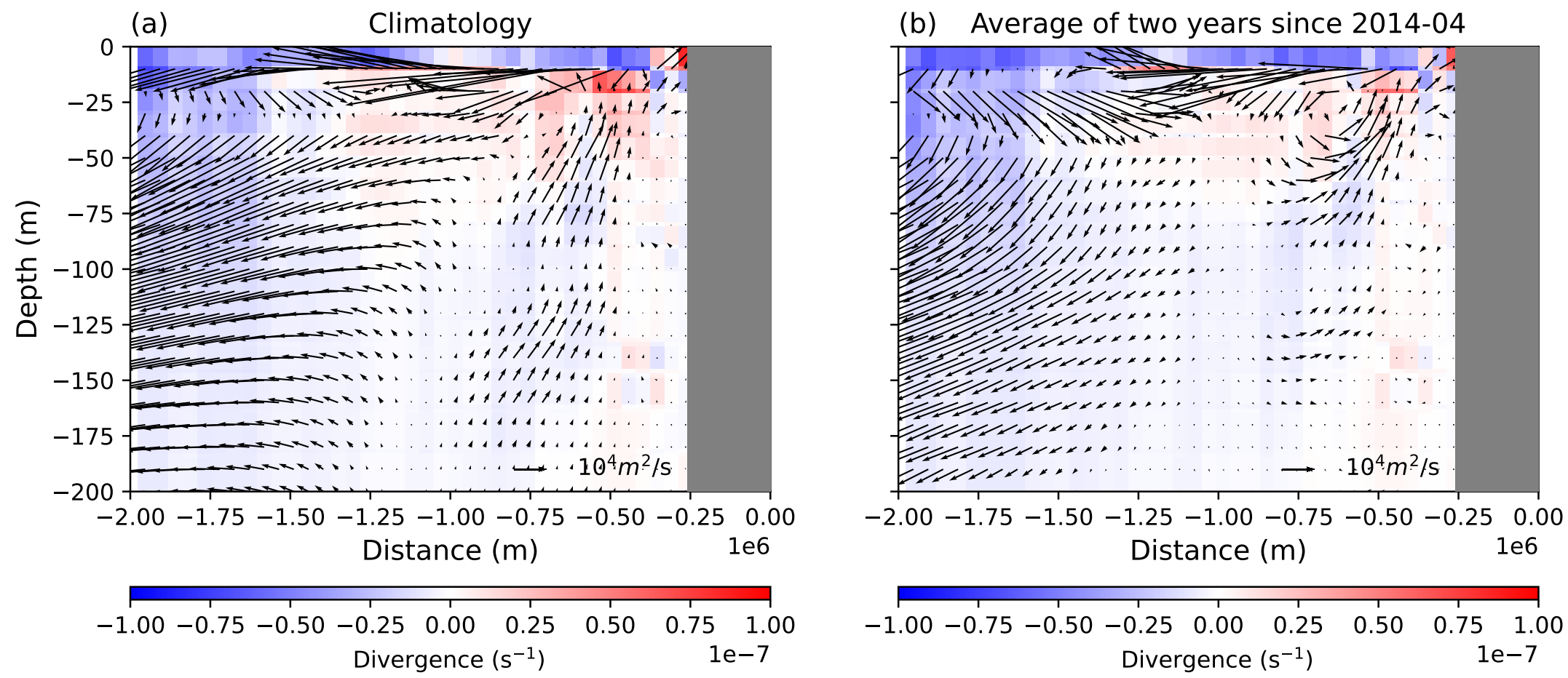

Figure R1: Overturning at CC. The arrows show the onshore and vertical component of the velocity integrated in the along-shore direction. Because of the large aspect ratio of the domain, the angles of the arrows are scaled the same way as the domain, i.e., the arrows are parallel to water parcel trajectories. The shadings are the velocity divergence of those two components.

We have added a sentence in section 4.3 after the equations to explain the source of the delta-term. It involves the correlation of temperature and velocity during each output period. Further discussion can be found in Jiang and Haine (2025).

3) How is the climatological cycle defined? Depending on the definition, Equations 2–3 may include additional terms. For example, if climatology is defined by filtering or smoothing in time, applying a bar operator to anomalies does not necessarily yield zero.

We have moved the definition of the overline operator in section 4.3 to make the definition more explicit. The climatological cycle is defined by the seasonal average during the ECCO period, i.e., averaging the first day of the year across all years, the second day of the year across all years, etc. This averaging has the property of $\overline{f} = \overline{\overline{f}}$. As a result, the mean of anomalies are always zero.

We have also checked the Eulerian mean of each anomaly term, and the residual of the temperature anomaly equation (Equation (3) in section 4.3). Both are as close to zero as the machine precision allows. You can find the notebook that checks these results in our GitHub repository, which is included in Open Research statement.

4) Vertically integrated meridional velocity is not, strictly speaking, a stream function, since zonal exchanges are not accounted for. Its contour lines do not correspond to overturning circulation unless the velocity is also integrated zonally from coast to coast. Please clarify the meaning of the "proxy stream function" in section 4.6. I am concerned that if the stream function is not properly calculated, the discussion in section 2.2 may be misleading. Why not present the two-dimensional Ekman transport and its convergence instead?

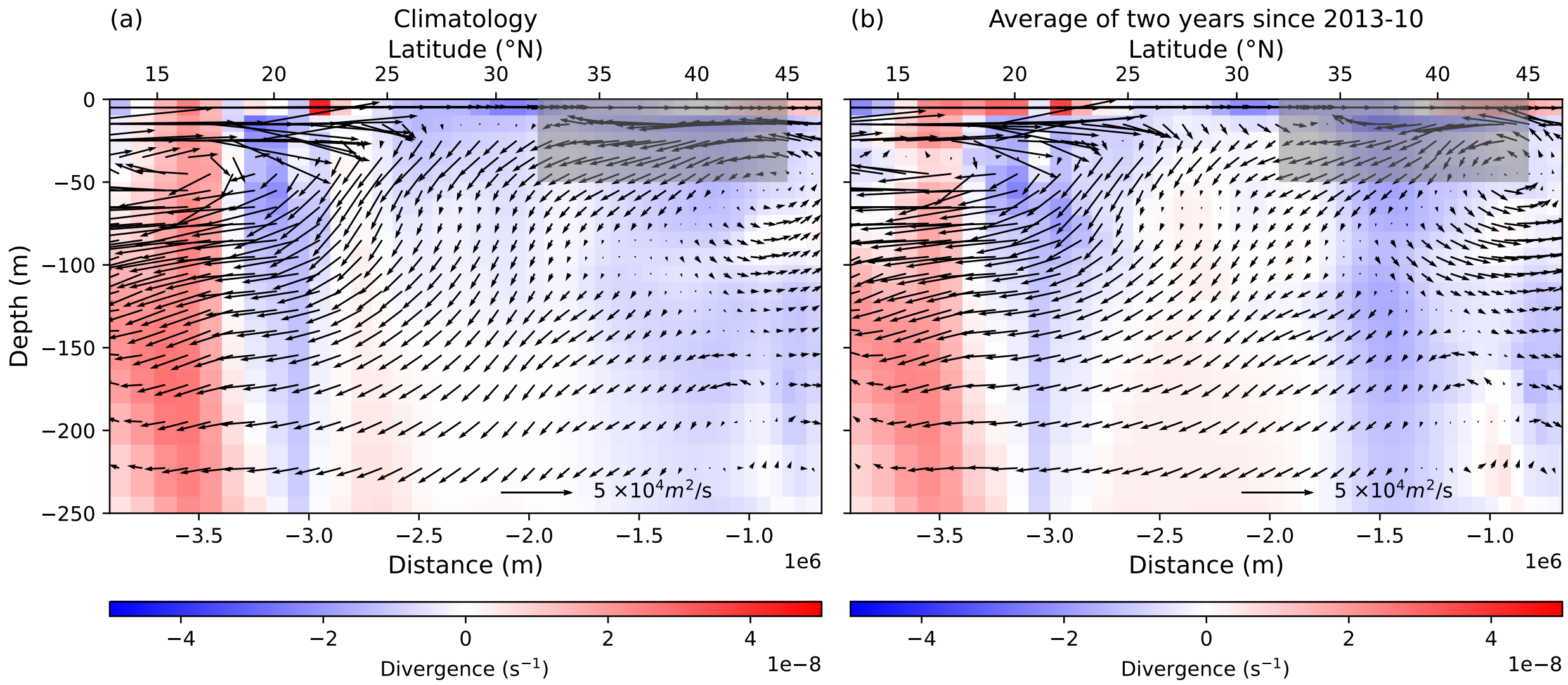

Figure R2: Same as Figure R1, but for GOA.

There is no perfect way to cast the time-variable three-dimensional ocean circulation in a two-dimensional plane. Here we discuss two different perspectives: (a) Horizontal plane: Figs. 4h and i show the Ekman transport in both MHW areas. For GOA, the dominant process is anomalous northward flow crossing the temperature front (Fig. 4h). For CC, the anomalous downwelling is because of the solid boundary. This convergence is shown by the arrows pointing towards land in Fig. 4i. (b) Vertical section: The new supplementary figures (Figures S7 and S8) show the overturning with quiver plots. In this response document, Figures R1 and R2 also show the same quiver plots overlaid on the convergence of zonal and along-shore velocity, respectively. The magnitudes of these convergences are very small.

Figures S7 and S8 show that the proxy-overturning captures well the patterns seen in the quiver plots. We decide not to use the quiver plot to replace the ones in the main figure for these reasons: First, because of the large aspect ratio of the ocean, the actual velocity vectors are mostly horizontal. To show overturning with the quiver plot, we need to rotate the vectors such that the arrows are parallel to water parcel trajectories . In this process, the magnitude of each component of the velocity is distorted. Second, the proxy-streamfunctions are integrated quantities that capture the main features seen in the quiver plots. Third, readers can estimate how the transport changed by comparing the proxy streamfunction contours, which is much harder with quiver plots. Finally, we find the quiver plot aesthetically less pleasing.

We have now added more explanation on why we chose the proxy stream function in section 4.6, with a reference to the new plots in Supplementary Materials to help the reader not get confused on these quantities.

5) Do surface winds explicitly depend on SST anomalies in this reanalysis? Could an increase in wind speed over warm SST anomalies, as described in the Vertical Mixing Mechanism (Wallace et al., 1989), be a significant factor in the lifecycle of the MHW?

Thanks for this great question. Yes, to a large degree surface winds explicitly depend on SST anomalies in this reanalysis. In ERA5 and MERRA2, SST plays an important role in driving the atmospheric dynamical model which predicts near surface wind speed. While ECCO does not explicitly resolve atmospheric dynamics above the sea surface, it inherits some of the coupling from MERRA2. In addition, ECCO4 uses bulk formulae to compute air-sea fluxes, to account for some of the feedbacks to SST, as is standard practice in forced ocean models.

The windspeeds over positive SST anomalies for the period we studied are often reduced or neutral (negative wind speed anomaly, e.g. Figs E8 and E10). However, it is possible that the Wallace et al. (1989) sequence of mechanisms (and also Spall, 2007) could play a role in the evolution of other MHWs. It is not easy to test this hypothesis though, and it is certainly beyond the scope of this paper.

Reviewer #2

General Comments

The authors investigate the mechanisms responsible for the anomalously prolonged marine heatwave (MHW) conditions in the northeastern Pacific Ocean during 2013–2016 using reanalysis products and OI-SST data. Given the strong influence of MHWs on marine ecosystems and their feedbacks to the overlying atmosphere, this study addresses a problem of considerable scientific and societal relevance. While the manuscript provides a coherent large-scale interpretation of the coupled air–sea processes sustaining the event, it does not sufficiently explore the seasonal evolution of the MHW. A more detailed examination of how the dominant physical processes vary across different seasons, such as winter versus summer contributions of surface heat fluxes, ocean heat transport, and mixed-layer processes, would substantially strengthen the present analysis. Therefore, I recommend that the manuscript be revised to include a more in-depth, seasonally resolved analysis of the MHW evolution, as mentioned in the comments below. With these revisions, the manuscript would be suitable for publication in Nature Communications. Hence, recommended for minor revisions before accepted for publication.

Thank you for your supportive assessment and helpful suggestion! Representing the MHW as a seasonal anomaly does mask the contribution of the **mean** seasonal cycle. We have added the mean seasonal cycle in Figure S3, and added some discussion in section 4.3 on why subtracting the mean seasonal cycle is useful to better reveal anomalies from it. Please refer to our responses below related to seasonality for additional information.

Other comments.

1) The authors mentioned that the ECCO data and OI-SST show good agreement in reproducing the MHW. However, it is unclear whether the performance of the ECCO model simulation with OISST in the NEP during the 2013-2016 study period. Hence, it is suggested to make a comparison

between OISSST and ECCO to demonstrate what we saw in the MHW conditions in the OISST is rightly reproduced in the ECCO data. Similarly, readers would like to know how well the simulation of ECCO reproduces the sub-surface information up to 150 m. Hence, a comparison with Argo floats in the NEP during 2013-16 is imperative.

For the comparison between OISST and ECCO, the end of the third paragraph in section 4.1 now reads: "Good agreement between OISST and ECCO4 for marine heat wave metrics was recently reported in Sala et al. (2025) (see also Figs. E1, E2), which also highlights the unique insights provided by ECCO4's closed heat budgets in this context." Sala et al. mainly compare the trend and seasonality in these two products, while Figs. E1, E2 compare the anomalies relative to the mean seasonal cycle.

We have added four new figures (Figs. S1, S2, S4, S5) in the supplementary material comparing the anomalies and seasonalities in ECCO4 and Argo, and added some brief discussion in the beginning of section 2. The agreement between ECCO4 and Argo is very good. This good agreement is also noted in the literature (e.g., Liao and Hoteit, 2022; Sala et al., 2025; Forget et al. 2015). We have also added references to published validation studies in the same sentence.

2) In the Results section. The northern anomaly and the southern anomalies. Please mark this area in the figure and specify this domain in the text.

The northern and the southern anomalies are both Lagrangian volumes, which means their spatial extent changes with time. The way we visualize the extent of those moving volumes is by showing the location of the particles (Figs. 1, E4–6). Please also see our response for comment No. 10.

3) In the Results section. The movement of the particle is mainly due to horizontal circulation. Hence, how can we connect that with marine heat wave propagation? I think a simple Hovmoller diagram of MHW intensity will provide insight into the spatial propagation of MHWs. Moreover, the spatial propagation or its westward extension can be associated with atmospheric flux impact.

To address this question we added an animation of particle positions and SST (see Supplementary Movie 1). This seems like the most effective approach because Hovmoller diagrams are restricted to one spatial dimension, whereas the water involved in the MHW takes many pathways even in the horizontal plane (i.e., even if we set aside the vertical component).

4) In the Results section. Though the authors provided SST anomalies, there is no information on whether these SST anomalies are classified as MHW. It is strongly recommended to prepare a figure for MHW for SST and OHC similar to Figure E1/E3. In addition, it is also recommended to add cumulative MHW intensity in each season.

Thank you very much for this great suggestion. We have now added a new figure (Fig. 2), a new supplementary figure (Fig. S6), a paragraph in the results section, and another paragraph in the methods section, all based on Hobday et al. (2016), which is the most commonly used method for classifying MHWs and calculating their cumulative intensity. We find that this addition helps

with the lack of seasonal analysis, fills in the gap of MHW classification in our paper, and also connects this paper to the broader literature.

5) 2014-12 and 2015-04, the SST anomaly cannot be considered as northern and southern MHW. Since it is apparent that a warm anomaly persists through the East Coast. Even in 2015-11, the MHW is clearly a single entity.

Yes, the contours of SST anomalies are indeed connected. In many senses, the reviewer is correct that the N-MHW and S-MHW can be viewed as a single entity. In light of the comment, we have now changed changed the terminology from "the northern MHW (N-MHW)" to "the northern part of the MHW (N-MHW)" to avoid implying a categorical separation.

Whether adjacent warm anomalies fundamentally constitute a single MHW or distinct events sometimes is not a very clear-cut question though. Recent tracking algorithms such as Ocetrac (Scannell et al., 2024) and SCMHW-CLUSTER (Yao et al., 2025) have shown that event boundaries depend sensitively on threshold choices for merging and splitting, which are subjective. Our Lagrangian approach sidesteps this ambiguity by tracking water masses directly: the particles constituting the northern and southern components originate in different regions, travel along different pathways, and are sustained by different processes. We find this distinction physically meaningful and well suited to our purpose of diagnosing the mechanisms driving each part of the MHW separately.

6) The Lagrangian-based framework adopted by the authors may be difficult to follow for a broad readership. To facilitate clearer interpretation, I suggest additionally performing a simplified heat budget for the upper 150 m of the water column and comparing the individual budget terms with their climatological counterparts. Such an analysis would help clarify which physical processes primarily contribute to the maintenance of the marine heatwave in each of the two boxes.

We agree that traditional Eulerian budgets are more familiar to most readers. Therefore, in the new Figure S3 we chose two large regions to represent the N-MHW and S-MHW to calculate the Eulerian heat budget (Figure S3 c,e). We added a reference to this figure in section 2.1. The budget results resemble Figs. 2 and 3, but are quantitatively different. The main reason for the difference is that not all the water included in the Eulerian budget is part of the Lagrangian budget.

7) In section 4.2, it is mentioned that K is the diapycnal and horizontal diffusivity. There is no information on the value of these constants that we used in the budget. Its values will be different both at the surface and the thermocline for vertical. How authros derived these informations.

Sorry for the confusion. The diffusivity in ECCO is a spatially and temporally varying field that is saved like any other field. For accuracy, we do not use diffusivity as part of the budget calculation. Instead, we use the diffusive flux calculated online in the model, which is the best way to close a heat budget. We have now explained this in more detail in section 4.3.

8) Section 2.1. Over time scale of few months. Which months. Please specifiy.

We are talking about variability in general. We have rephased to “Temperature variability operates on different timescales. On shorter timescales (e.g., a few months, as seen between December 2014 and April 2015)...”.

9) Section 2.2. later 2014. The cumulative heat flux shows large heat loss. From Figure 2a.

Yes, we have stated in section 2.2 that there is a large heat loss. Note: there seems to be a formatting issue that cut out the question here.

10) I am not sure whether the authors performed the budget with respect to the 150 m or the mixed layer. If it is the first case, the heat flux impact is mainly restricted to the upper 30-40 m of the water column. Please specify.

As noted right before section 2.1, the Lagrangian budget is performed in a Lagrangian volume: “Notably, N-MHW and S-MHW represent time-evolving Lagrangian volumes that record the history of water parcels, which differ conceptually from common definitions of MHWs based on an instantaneous temperature threshold.”. After that sentence, we have now added: “Heat budget results on N-MHW and S-MHW are also conceptually different from that defined in a mixed-layer or a fixed volume.” We close the temperature budget of each particle, and integrate the particles to describe the overall heat budget. This is discussed in detail in the Method section. In more concrete terms, the budget is conducted on all the water parcels that have been part of the MHW, wherever they may be at other times.

11) Figure 2b and 2c; what is the justification for selecting a different period for constructing mean picture.

The periods are chosen because they represent the first growth and mature/decay periods. The first sentence of the second paragraph of section 2.1 now says: “Fig. 3b–i show how anomalous advection and surface heating affect the N-MHW spatially during each growth (predominantly warming) or maturity/decay (steady or cooling) periods.” (Here, Fig. 3 is the original Fig. 2).

Reviewer #3

This study investigates the 2013–2016 marine heatwave (MHW) events in the northeastern Pacific Ocean. The authors employ a Lagrangian heat budget framework to track the evolution of these events and diagnose the dominant physical processes governing their development and persistence. The analysis is based primarily on the ECCO v4 release 4 global ocean state estimate. A series of passive tracer experiments are conducted using the ECCO Modeling Utilities (EMU) under no-flux boundary conditions. Observational and atmospheric reanalysis products, including OISST and ERA5, are used to qualitatively support several aspects of the results. The study highlights the roles of Ekman heat transport and Ekman upwelling in shaping the SST anomalies and further links the events to large-scale climate modes such as the Pacific Decadal Oscillation (PDO) and variability associated with the Aleutian Low. These mechanisms are summarized in a clear and well-designed schematic diagram.

Despite these strengths, I have two major concerns regarding the novelty and methodological suitability of the study.

1) The 2013–2016 northeastern Pacific marine heatwave has been extensively documented in the literature, with numerous studies already describing its physical drivers, air–sea coupling, and large-scale climate connections. While the present study applies the ECCO framework and a Lagrangian heat budget approach, the resulting physical interpretation largely reiterates mechanisms that are already well established, including reduced heat loss, anomalous Ekman transport, and basin-scale atmospheric forcing. The manuscript does not clearly articulate what fundamentally new insight is gained beyond re-diagnosing known processes using a different modeling framework. In addition, it is surprising that the study does not explicitly reference the term "California Blob," which has been widely used in prior literature to describe the 2013–2016 event and provides important historical and conceptual context. The absence of this terminology, without justification, further obscures the connection between this work and the existing body of research.

We thank the reviewer for this comment, which has prompted us to sharpen the presentation of our innovative contributions. We respectfully but firmly disagree that this work reiterates well established mechanisms, and we address this point-by-point below.

First, the vast majority of prior studies focus on the *initiation* of this event in the winter of 2013 (e.g., Bond et al. 2015, Shi et al. 2024, Chen et al. 2023, Chen et al. 2025). By contrast, our Lagrangian framework tracks the MHW through its entire life cycle—from emergence through intensification to decay—and quantifies the contribution of each process at every stage. Our analysis of heat content anomaly throughout the life cycle is, to our knowledge, without precedent for this event.

Second, the reviewer correctly notes that reduced surface heat loss and anomalous Ekman transport have been identified as plausible contributing mechanisms by leveraging disparate estimates to infer parts of the MHW heat budget. However, this is fundamentally different from quantifying mechanisms within a closed, definitive, and integrative budget estimate where all the terms are included and there is no disagreement between them.

Our framework answers *how much*, *where*, and *when* each process contributes—questions that cannot be addressed by the existing literature. For example, we show that advective processes, rather than surface forcing, play the quantitatively dominant role in sustaining the MHW's heat content—a result that challenges the emphasis of several prior studies (e.g., Schmeisser et al. 2019). We also identify the weakening of Ekman heat transport specifically across the North Pacific Current temperature front as the single largest contributor to the growth of the N-MHW, and quantify its magnitude—a level of specificity that previous studies failed to reach.

Third, it is true that the individual links between SST and SLP have been explored in isolation (e.g., Ji et al. 2023, Amaya et al. 2020, Hartmann 2015) and atmospheric forcing has been implicated in the MHW's persistence (e.g., Bond et al. 2015, Di Lorenzo and Mantua 2016). But no prior study has provided a quantitative, process-resolved budget; and no prior study has closed the positive feedback loop in a quantitative, holistic way. Our Lagrangian budget allows us to show

that the SLP anomaly more than compensates for the heat lost by the MHW to the atmosphere, completing the loop and providing a mechanistic explanation for the event's unusual multi-year persistence.

Fourth, our three-dimensional particle trajectories reveal that the northern and southern components of the MHW are kinematically independent—they do not share a common water mass origin. This finding has not been reported before and fundamentally reframes the Blob as being made of two dynamically distinct water bodies under a shared atmospheric driver, rather than a single coherent SST feature.

Finally, beyond the specific findings for this event, we emphasize that defining MHWs using a closed Lagrangian heat budget framework in itself represents a paradigm shift for the MHW community that has largely focused on statistical detection algorithms for extreme events and SST anomaly kinematics.

This is the first time such a framework has been formulated and demonstrated for MHWs. The approach is general: it can be applied to any MHW event, and it is particularly well suited to large-scale, propagating warm anomalies where the fundamentally advective nature of the event makes Eulerian analyses incomplete and generally inadequate. The 2013–2016 Blob was chosen for this first application of our new framework because of its prominence and the wealth of prior research available for context, but the framework is readily applicable to other major events and future MHWs as they emerge. We believe the generality and solid theoretical underpinning of the novel framework—a reusable, objective, and dynamically consistent diagnostic framework—are a significant advance, apart from the novel insights it yields for the 2013—2016 event.

To ensure these contributions are immediately apparent, we have revised the abstract, the last paragraph of the introduction, and the discussion to highlight the novelty more explicitly.

2) A more serious concern relates to the suitability of ECCO for studying marine heatwaves as they are formally defined. Marine heatwaves are typically identified as periods during which SST exceeds the 90th percentile of a climatological baseline for at least five consecutive days, following the definition of Hobday et al. (2016). ECCO state estimates are optimized to provide dynamically consistent, global ocean reconstructions that are most reliable at monthly to longer timescales. As such, ECCO is not specifically designed to resolve short-duration extreme events at the temporal precision required for MHW detection and characterization. This limitation raises questions about whether ECCO can robustly capture the onset, peak intensity, and duration of marine heatwaves as defined by percentile-based, daily SST thresholds. While ECCO may be useful for diagnosing large-scale heat transport and low-frequency variability, its application to event-scale marine heatwave analysis requires stronger justification and careful discussion of uncertainties.

Thanks for raising these very important points. There are two main parts to the reviewer's concerns: (1) ECCO4 is not suitable for the Hobday et al. approach. (2) If the Hobday et al. approach is not used, one cannot discuss MHW as an event.

Although we did not use the Hobday et al. definition in the initial submission, we have now added new analysis on MHW identification based on both daily mean ECCO (Fig. S6) and daily

mean OISST (Fig.2). The results are very consistent between the two estimates.

We find that this comparison is helpful in locating our study in the context of the existing literature. Since the daily mean OISST product is commonly used for this kind of analysis, the similarity between the diagnostics based on the two products demonstrates that ECCO can effectively capture MHW characteristics consistent with the standard approach. In addition, we refer the reviewer and reader to Sala et al. (2025) for a nice overview of MHW detection with ECCO. This, we hope, can address the first part of the reviewer's concern.

We tend to disagree with the reviewer's second claim, because it seems to rule out the possibility of using low temporal and spatial resolution in situ observations to study MHWs. Argo, for example, has a resolution of one profile every ten days and 300 km. The reviewer's argument implies that Argo is inadequate to study marine heatwaves because it does not have the resolution of the daily SST dataset used in Hobday et al. (2016). We tend to disagree and want to emphasize that when we are studying the heat content rather than just the surface signature of a MHW, Argo is the best observational dataset that has consistent global coverage. Being constrained by Argo is a key reason why ECCO is advertised to be most useful at monthly and longer time scales. Among gridded Argo products though, ECCO may in fact be best equipped to interpolate between observations, owing to its use of ocean dynamics as encoded in MITgcm and additional data constraints from satellite. Furthermore, the events and time scales being considered in this paper are multi-seasonal and basin-scale. None of our results depend on resolving the detailed time evolution of events at daily time scales. The shortest time scale we discuss in the paper is the rapid winter warming in the winter between 2014 and 2015, which lasts about 3 months.

A key innovation of our study is to introduce a more mechanistic concept and method that tracks the large body of North Pacific water that experienced the MHW events of 2013–2016 while moving through the domain. This is fundamentally different from tracking temperature anomalies from a kinematic point of view using SST anomalies or examining heat budgets on a fixed grid.

Overall, while the study is clearly written and methodologically thorough within the ECCO framework, the lack of clear novelty and the potential mismatch between the chosen model product and the temporal definition of marine heatwaves substantially weaken the impact of the manuscript. Addressing these concerns, by clarifying the unique contributions of the study and more rigorously justifying the use of ECCO for MHW analysis, would be essential before the work can be considered for publication.

We thank the reviewer for acknowledging the clarity of the writing and the methodological thoroughness of the analysis. Regarding the temporal definition of MHWs, we note that our study does not rely on the Hobday et al. definition; our MHW identification is based on the Lagrangian heat content anomaly, as described in Methods. We therefore believe this concern does not apply to our analysis. Regarding novelty, we hope that the point-by-point response above—together with the revised abstract, introduction, and discussion—has made clear that this work offers several contributions that are distinct from the existing literature, both in methodology and in physical insight.
